# A Shrewd Inspection of Vertebral Regionalization in Large Shrews (Soricidae: Crocidurinae)

**DOI:** 10.1093/iob/obac006

**Published:** 2022-02-10

**Authors:** Stephanie M Smith, Kenneth D Angielczyk

**Affiliations:** Field Museum of Natural History, Negaunee Integrative Research Center, 1400 S DuSable Lake Shore Drive, Chicago IL 60605, USA; Field Museum of Natural History, Negaunee Integrative Research Center, 1400 S DuSable Lake Shore Drive, Chicago IL 60605, USA

## Abstract

The regionalization of the mammalian spinal column is an important evolutionary, developmental, and functional hallmark of the clade. Vertebral column regions are usually defined using transitions in external bone morphology, such as the presence of transverse foraminae or rib facets, or measurements of vertebral shape. Yet the internal structure of vertebrae, specifically the trabecular (spongy) bone, plays an important role in vertebral function, and is subject to the same variety of selective, functional, and developmental influences as external bone morphology. Here, we investigated regionalization of external and trabecular bone morphology in the vertebral column of a group of shrews (family Soricidae). The primary goals of this study were to: (1) determine if vertebral trabecular bone morphology is regionalized in large shrews, and if so, in what configuration relative to external morphology; (2) assess correlations between trabecular bone regionalization and functional or developmental influences; and (3) determine if external and trabecular bone regionalization patterns provide clues about the function of the highly modified spinal column of the hero shrew *Scutisorex.* Trabecular bone is regionalized along the soricid vertebral column, but the configuration of trabecular bone regions does not match that of the external vertebral morphology, and is less consistent across individuals and species. The cervical region has the most distinct and consistent trabecular bone morphology, with dense trabeculae indicative of the ability to withstand forces in a variety of directions. *Scutisorex* exhibits an additional external morphology region compared to unmodified shrews, but this region does not correspond to a change in trabecular architecture. Although trabecular bone architecture is regionalized along the soricid vertebral column, and this regionalization is potentially related to bone functional adaptation, there are likely aspects of vertebral functional regionalization that are not detectable using trabecular bone morphology. For example, the external morphology of the *Scutisorex* lumbar spine shows signs of an extra functional region that is not apparent in trabecular bone analyses. It is possible that body size and locomotor mode affect the degree to which function is manifest in trabecular bone, and broader study across mammalian size and ecology is warranted to understand the relationship between trabecular bone morphology and other measures of vertebral function such as intervertebral range of motion.

## Introduction

Increased regionalization and heterogeneity of the vertebral column are hallmarks of mammals and emerged deep in their evolutionary history (Buchholtz 2014; Jones et al. 2018; Jones et al. 2019). Understanding the evolution of morphological regionalization in the mammalian spine is critical to tracking the evolution of mammalian gaits, locomotor diversity, and respiratory function (Gál 1993; Boszczyk et al. 2001; Schilling and Hackert 2006; Vander Linden et al. 2019; Jones et al. 2021). Because the spinal column provides support for the viscera and skull, anchor points for the appendicular skeleton and skeletal muscles, and a flexible but protective conduit for the spinal cord, it is central to mammalian motion and behavior throughout ontogeny. This means that vertebral morphology is subject to a variety of different evolutionary pressures and constraints (Asher et al. 2011; Jones et al. 2018). Developmentally, primary patterning of the regionalized vertebral column is controlled by highly conserved *Hox* genes (Wellik 2007; Wellik 2009; Hautier et al. 2010; Mallo et al. 2010; Head and Polly 2015; Böhmer 2017). Recent work (Jones et al. 2020) indicates that the evolution of developmentally-defined regions probably preceded functional disparity among these regions, and that morphological differentiation is therefore a precondition for functional diversification in the vertebral column.

Investigations of functional regionalization in the vertebral columns of mammals and their non-mammalian synapsid ancestors have so far focused on traits that are directly related to gross vertebral morphology, including intervertebral joint mobility/range of motion and morphological integration and modularity (Randau et al. 2016; Randau and Goswami 2017; Jones et al. 2020; Belyaev et al. 2021; Martín-Serra et al. 2021; Figueirido et al. 2021). However, bone functional capabilities relate not only to gross morphology, but also to bone microstructure, because trabecular (cancellous) and cortical (compact) bone share and respond to in-vivo loads together (Silva et al. 1997; Eswaran et al. 2006; Fields et al. 2009). Trabecular bone architecture (TBA) is quantitatively related to the magnitude and direction of in-vivo forces acting on the whole bone (Cowin 1986; Wolff 1893; Ruff et al. 2006; Kivell 2016), meaning that TBA can provide a snapshot of the mechanical environment acting on the skeleton of the animal in question.

To further elucidate the connections among development, morphology, and function in the mammalian vertebral column, we set out to examine a potential new important but underinvestigated aspect of vertebral regionalization: craniocaudal variation in the structure of vertebral trabecular bone. This topic is of interest because it has the potential to uncover the relationship between developmentally-determined gross morphology regions and potentially more plastic regions defined by trabecular bone variation. Additionally, the evolution of new functional regions in the mammalian spinal column has been shown to follow developmental regionalization in synapsid evolution (Jones et al. 2020), but the relationship between TBA and external-morphology functional regions has not yet been examined. An understanding of morphofunctional regionalization across anatomical scales could also give clues about the origin and purpose of more recent evolutionary novelties within mammals, including the famously unique and enigmatic spinal column of the hero shrew *Scutisorex*, which we consider here. In the interest of investigating the *Scutisorex* vertebral column in a meaningful morphological context, we chose to focus this study on relatively large shrews (body mass ∼30–80 g) of the subfamily Crocidurinae. The functional morphology of *Scutisorex* is notoriously difficult to study because its highly divergent vertebral shape confounds homology-based morphometric approaches (Smith and Angielczyk 2020). Here, we employ a combination of homology-based gross morphology and homology-free trabecular bone morphology to address the following primary research questions:

1. Do the spinal columns of large crocidurine shrews exhibit microstructurally-defined regions in addition to those defined by gross morphology, and if so, what clues can these regions give us about how development and function influence vertebral morphology?
2. Does regionalization of gross or trabecular bone morphology differ between *Scutisorex* and other unmodified shrews? If so, could those differences give us hints into the in-vivo use of the unusual *Scutisorex* spine?

## Methods

### Specimen selection and µCT scanning

We selected at least two males and two females of three species of large shrews from the mammalogy collections of the Field Museum of Natural History (FMNH) to include in our analyses. The fourth species, *Scutisorex thori*, is represented by three specimens, which is the entire known sample of the species (Smith and Angielczyk 2020). The similar sizes of the four species (Table 1) mitigate the effects of body size on our analyses; we report body mass from FMNH field notes for each specimen (Table 1). All specimens are adults based on fusion of long bone epiphyses. The total sample is *n* = 19 specimens, including both dry skeletons and alcohol-preserved carcasses as noted in Table 1.

**Table 1:** Specimens included in the current study. FMNH 223983 (denoted with an asterisk), has one additional transitional lumbar-sacral vertebra, which is fused to the first sacral and was therefore not included. Holotype of *Scutisorex thori* denoted with a double asterisk. Total = total presacral number, including third cervical (C03) through last lumbar. Diaph = diaphragmatic position, For example, 20 (T15) means that the diaphragmatic vertebra is position 20 in the overall presacral column, corresponding to the fifteenth thoracic position.

Specimen No.	Taxon	Prep type	Sex	Mass (g)	Thoracic count	Diaph.	Lumbar count	Total
FMNH 162144	*Crocidura goliath*	alc	F	58	14	17 (T12)	5	24
FMNH 162185	*Crocidura goliath*	dry	M	51	14	17 (T12)	5	25
FMNH 162186	*Crocidura goliath*	dry	F	52	14	17 (T12)	5	24
FMNH 167691	*Crocidura goliath*	alc	M	57	15	18 (T13)	5	24
FMNH 213932	*Suncus murinus*	alc	M	37	15	18 (T13)	5	25
FMNH 213935	*Suncus murinus*	alc	F	51	14	18 (T13)	5	25
FMNH 213944	*Suncus murinus*	alc	M	27	15	18 (T13)	5	25
FMNH 213945	*Suncus murinus*	alc	F	22	14	18 (T13)	5	25
FMNH 137613	*Scutisorex somereni*	dry	M	67	13	18 (T13)	11	29
FMNH 148270	*Scutisorex somereni*	dry	?	59	13	18 (T13)	12	30
FMNH 148271	*Scutisorex somereni*	dry	F	69	14	18 (T13)	11	30
FMNH 148941	*Scutisorex somereni*	dry	F	65.5	14	18 (T13)	11	30
FMNH 160178	*Scutisorex somereni*	dry	M	54.5	13	17 (T12)	11	29
FMNH 160180	*Scutisorex somereni*	dry	M	44	13	17 (T12)	11	29
FMNH 223983*	*Scutisorex somereni*	alc	M	81	13	17 (T12)	10*	28
FMNH 227556	*Scutisorex somereni*	alc	F	76	13	17 (T12)	11	29
FMNH 219669**	*Scutisorex thori*	dry	F	47	14	16 (T11)	8	27
FMNH 222612	*Scutisorex thori*	alc	M	36	14	16 (T11)	8	27
FMNH 222613	*Scutisorex thori*	alc	M	49	14	17 (T12)	8	27

All specimens were scanned in the GE v|tome|x µCT scanner at the University of Chicago PaleoCT facility. Scan resolution ranged from 14–26 µm. We captured the entire presacral vertebral column of each specimen, which in most cases required multiple scans. When cranial and caudal sections of the column were scanned separately, we minimized the difference in resolution between the scans to reduce intraspecimen variation in quality (see Table S1). Four specimens had a difference of > 5 µm between the two scans (Table S1), which we considered carefully during trabecular bone segmentation in order to mitigate error introduced by partial volume effects (see below, Model Training and VOI segmentation). We reconstructed scans in GE phoenix datos|x and aligned and cropped the resulting image stacks using VGStudioMAX 3.3 (Volume Graphics, 2019).

### External vertebral measurements

**Fig. 1:**
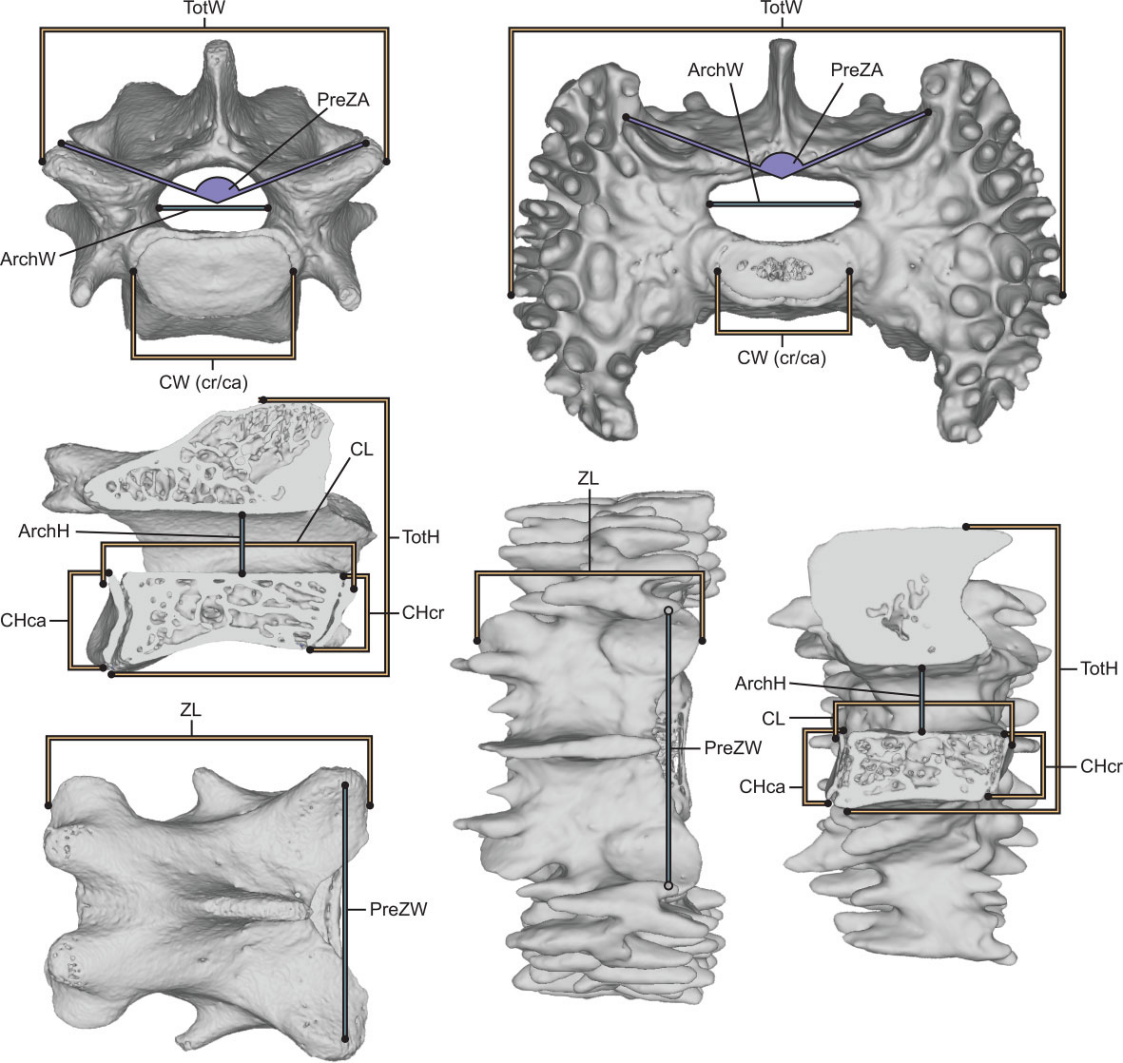
Linear measurements taken on all vertebrae in our sample, modified from Jones et al. (2018, Fig. S1, Table S3). Measurements are shown on L03 of FMNH 213935 (*Suncus murinus*; left)), and L06 of FMNH 148271 (*Scutisorex somereni*; right). Abbreviations (after Jones et al. 2018): ArchH = neural arch height; ArchW = neural arch width; CH (cr/ca) = centrum height (cranial/caudal); CL = centrum length; CW (cr/ca) = centrum width (cranial/caudal); PreZA = pre-zygapophyseal angle; PreZW = pre-zygapophyseal width; TotH = total height; TotW = total width; ZL = zygapophyseal length.

We imported reconstructed image stacks into ORS Dragonfly (version 2021.1) and segmented via gray value thresholding to produce 3D surface models of each vertebra, starting with the third cervical position and extending caudally to the last lumbar position. We did not include the atlas and axis because their morphologies are fundamentally different from the rest of the presacral vertebrae, and therefore difficult to quantify and compare using homologous measurements. After exporting vertebral models as .STL files, we took 12 linear and angular measurements on each one (Fig. 1), using a combination of the “Units/dimensions” tool in Autodesk Meshmixer (version 3.5.474) and the “Markups” module of 3DSlicer (version 4.11 (Fedorov et al. 2012)). We customized the measurement scheme of Jones et al. 2018 to include only measurements that could be accurately obtained on both the modified vertebrae of *Scutisorex* and on the more typical vertebrae of *S. murinus* and *C. goliath*.

### Volume of interest (VOI) selection

**Fig. 2:**
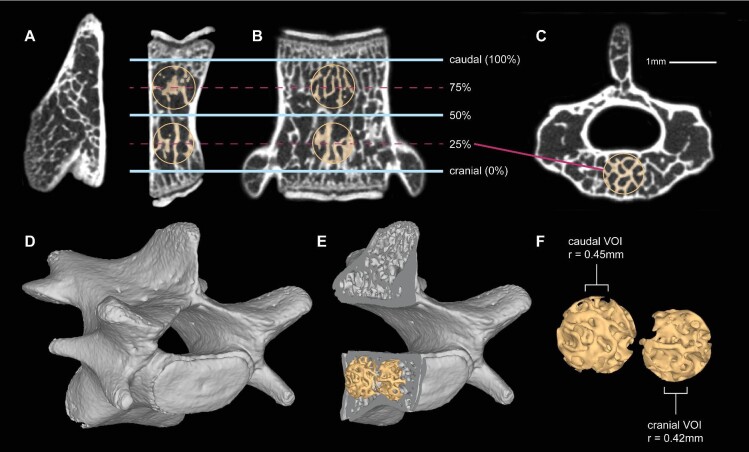
Determination of VOI size and location in vertebral centrum. A–C: reconstructed sections through the third lumbar vertebra (L03) of FMNH 213935, *Suncus murinus* (A = sagittal; B = frontal; C = transverse). Cranial and caudal VOIs were centered at 25 and 75%, respectively, of the craniocaudal distance between the first and last transverse slices containing no evidence of the epiphyses (A, B). VOI size was limited in most cases by the dorsoventral dimensions of the vertebral centrum (visible in A), and in all other cases was constrained by the cranialmost or caudalmost slice and the slice at 50% of craniocaudal length (B). D–F: 3D reconstructions of whole vertebra (D), sagittal section of vertebra showing location of 3D VOIs (E), and detail of VOIs, each with its radius listed in mm (F).

To sample the maximum quantity of trabecular bone inside a vertebra while maintaining a standard VOI shape, we selected two spherical VOIs: one cranial and one caudal (Fig. 2). We determined the placement and size of these VOIs following the method of Fajardo et al. 2013. After loading reconstructed image slices into ORS Dragonfly, we separated each vertebra into its own uniformly oriented substack, with the image slices in the transverse plane along the craniocaudal axis of the vertebral centrum. To establish the bounds of the centrum's trabecular bone, we searched through the stack and marked the first (cranialmost) and last (caudalmost) slice capturing trabecular bone but no evidence of the cranial or caudal epiphyses. We centered the sphere of the cranial VOI at the slice 25% of the distance between the cranial- and caudalmost slices, and the sphere of the caudal VOI at the 75% mark. After centering the spheres, we expanded the diameter of each as far as possible without impinging on the cortical shell of the vertebra, the first or last trabecular slice, or the 50% slice. We positioned each sphere mediolaterally on the vertebral midline, and dorsoventrally to allow it to include the largest possible volume of trabecular bone. The resulting spheres were used to mask image stacks for the relevant vertebra, which were subjected to the segmenting process described below.

### Model training and VOI segmentation with deep learning neural networks

Our specimens were sourced from natural history collections and therefore varied in body configuration (e.g., spine curved or straight) and method of preservation (e.g., dry or fluid-preserved), so we could not use identical processing parameters for every specimen throughout our entire workflow. Previous work (Christiansen 2016; Smith and Angielczyk 2020) has shown that threshold-based segmentation can be subjective and result in a high degree of error in trabecular bone measurements, particularly bone volume fraction (BV.TV) and trabecular thickness (Tb.Th), especially in conjunction with variations in scan resolution. Furthermore, small and thin structures are difficult to segment, especially in noisy images (Van Aarle et al. 2014; Andrew 2018). To decrease the degree of error introduced by subjectivity of threshold selection while accounting for specimen variation and resulting scan quality, we chose to segment the extremely thin trabecular structures of the shrews using UNet Deep Learning models, which we trained using ORS Dragonfly's Deep Learning toolkit.

Machine learning segmentation techniques have been demonstrated to result in fewer misclassified pixels than both Otsu and watershed methods, and to perform well on noisy images (Andrew 2018; Dunmore et al. 2018; O'Mahoney et al. 2019). We trained two different UNet deep learning segmentation models to apply to our scans, each using a scan of a specimen from our dataset (FMNH 148271, *Scutisorex somereni;* FMNH 162186, *Crocidura goliath*). Each training data set started with six slices (two in each of three orthogonal 3D planes) in which we manually segmented out bone from background. We then iteratively trained UNet models with the ORS Dragonfly Deep Learning Tool for 50–100 epochs with the following parameters: patch size = 32 or 48; stride ratio = 0.75; batch size = 32. We continued training until the model was able to maximize selection of bone and minimize selection of background pixels, based on visual comparison of the training data set, the original scan, and the model-based segmentations.

After training the models, we tested them on a representative slice from each  µCT scan in our data set, and selected the best-performing model for a given scan to segment the VOIs in that scan. Because the two models (148271 and 162186) were trained on different specimens, they have some small differences, and usually one performed slightly better than the other on a given scan. For more detailed comparisons of the performance of the two models, their performance relative to threshold-based segmenting, and information on model choice for segmenting each scan, see the electronic supplementary material (Figs. S1–S4). UNet models used here are available for download (see Data Availability Statement). We segmented spherical trabecular bone VOIs in Dragonfly using the “segment with AI” tool, and exported the resulting ROIs as binary image stacks with isotropic pixel spacing.

### Trabecular bone analysis

We used Quant3D (Ketcham and Ryan 2004; Ketcham 2005) to collect four trabecular bone metrics on each VOI: bone volume fraction (BV.TV), trabecular thickness (Tb.Th), trabecular number (Tb.N), and degree of anisotropy determined via mean intercept length (MIL.DA). Quant3D settings were as follows for all analyses: user-defined threshold of 127–255 (appropriate for pre-binarized images); 2049 uniform rotations; dense vectors on; random rotations on; omit side intersecting paths on; star volume distribution (SVD)/star length distribution calculated with 2000 points.

### Scan resolution, relative resolution, and the assumption of continuity

The trabecular elements in the sampled taxa can be extremely thin, and therefore difficult to detect, even in high-resolution scans. For example, the mean trabecular thickness in one of our VOIs was 26 µm in a scan with 12 µm resolution, so many structures are just one or two pixels wide. Previous workers have suggested that relative resolution (number of pixels per mean trabecular thickness, expressed here as px/tb; (Sode et al. 2008) should be at least five for a trabecular bone volume (Sode et al. 2008; Kivell et al. 2011), as lower-resolution scans make thin structures more susceptible to partial volume effects during segmentation (Soret and Bacharach 2007).

Mean relative resolution for all specimens (*n* = 1022 VOIs), as well as each species and each specimen, are listed in Table S2. According to Tb.Th measurements we collected using Quant3D, overall mean relative resolution in this study was 2.64 px/tb, which does not meet the resolution recommendations discussed above. However, the method Quant3D uses to calculate Tb.Th is based on the SVD (Cruz-Orive et al. 1992; Smit et al. 1998; Ryan and Ketcham 2002; Ketcham and Ryan 2004; Ketcham 2005), which represents average distribution of material around a typical point in a structure (Cruz-Orive et al. 1992). SVD is fundamentally different from the thickness-mapping method employed in the FIJI plugin BoneJ (Hildebrand and Rüegsegger 1997; Dougherty and Kunzelmann 2007; Doube et al. 2010). Several recent studies have used BoneJ Tb.Th measurements to demonstrate that their scan resolution is sufficient (e.g., Mielke et al. 2018, 7.78px/tb; Amson et al. 2017, 6.29 px/tb). For comparison to these studies, we calculated Tb.Th in pixels for all our VOIs, using BoneJ and removing scale from the images before calculation (Analyze > Set Scale > Remove Scale). This process yielded a mean Tb.Th of 4.38 px/tb (Table S2), a value 40% higher than the “equivalent” statistic calculated with Quant3D. The first use of the SVD-based Tb.Th calculation is likely from Ryan and Ketcham (Ryan and Ketcham 2002). That study noted that the SVD-based method is “not necessarily as robust an indicator of trabecular thickness as other methods” (p. 256), and cited as an example Hildebrand and Rüegsegger (Hildebrand and Rüegsegger 1997), which was used to formulate the method used in BoneJ. It is critical to note the difference between the results of these two Tb.Th methods when comparing Tb.Th and relative resolution among trabecular bone studies. A thorough comparison of the merits of the two Tb.Th measurements is beyond the scope of this paper, but should be investigated in the future to better inform trabecular bone study design.

Although our mean relative resolution is lower than that of other recent studies, even when determined using the BoneJ method, the papers mentioned above include animals that are mostly larger than the shrews considered here. This means they have thicker trabeculae (Doube et al. 2011) and require lower-resolution scans to obtain the recommended relative resolution. Our relative resolution is not unreasonably low, especially considering the small size of our animals and associated difficulties with obtaining sufficient scan resolution. Scale also hampers our ability to fulfill the guidelines for assumption of material continuity in trabecular bone (VOI/MIL ≥ 5; Harrigan et al. 1988). Even when selecting the entire width of the trabecular bone cavity, most VOIs in our study are smaller than five times the MIL due to absolute anatomical size constraints. However, our mean VOI/MIL is 4.28, which is close to guidelines in Harrigan et al. 1988 and is the best we can achieve with vertebral VOIs in these animals.

### Vertebral regionalization analyses

To assess number and location of morphological region breaks in the presacral vertebral column of our shrews, we used the segmented regression approach of Head and Polly 2015 and the regions package developed by Jones et al. 2018 (https://github.com/katrinajones/regions) implemented in R version 4.0.2 (R Core Team 2020). We modeled our protocol after the tutorial available at https://www.katrinaejones.com/code, and conducted separate analyses for the external measurement data and the TBA data. First, we executed principal coordinates analysis (PCO) using the Gower distance metric on a scaled version of our data set (regions function svdPCO, metric = “gower”). Next, we used the compileregions function to execute segmented regression analysis on PCOs 1–5, which for our sample included *at least* all PCOs individually accounting for 5% or more of total variation in the dataset. We decreased the number of PCOs considered here from 10, the number used in the tutorial, to reduce our already considerable calculation time (see below). To determine the most likely region configuration, that is, location and number of region breaks along the presacral spine, we compared the results of compileregions using the corrected Akaike Information Criterion (AICc) and log likelihood. We calculated region configuration using the function modelselect, considering *only* PCOs individually accounting for greater than 5% of total variation in the data (usually 3 or 4 coordinates).

The original version of the compileregions function was written to detect a maximum of 6 vertebral regions because considering a greater number of regions massively increases the number of possible region configurations, and results in a cumbersome increase in calculation time (K. E. Jones, pers. comm.). However, with the possible number of regions set to 6, several of our external morphology analyses resulted in an average number of regions >5.99. To allow for the possibility that a seventh region might be present, we wrote an alternate version of the compileregions function that allowed us to consider seven regions. This version of the function, as well as versions of several other regions functions that we edited for compatibility with the 7-region version of compileregions, are available on the regions GitHub repository (https://github.com/katrinajones/regions). The option for detecting seven regions will also be available in a future update of the regions package.

### Whole-column and between-region heterogeneity

We calculated whole-column heterogeneity for both external measurements and TBA following the methods of Jones et al. 2018 and Head and Polly 2015. We log-transformed the raw measurements for each data set, calculated the variance of each measure across the whole column for each specimen, and then calculated the mean of variances across all measures. This resulted in two heterogeneity values for each full vertebral column: one for external morphology, and one for TBA. We also assessed heterogeneity of regions *within* each column, that is, the degree of morphological disparity between adjacent regions in the same column. To do this, we used scaled data (function scale in base R) and the function daisy from the R package cluster (Maechler et al. 2012) to calculate the Gower distance matrix for all vertebral positions along the column of a given specimen. We then divided the column into regions based on the best supported configuration of region breaks as determined using the modelselect function (above), and calculated the distance between centroids of adjacent regions using the dist_between_centroids function from the package usedist (Bittinger and Bittinger 2017). Differences among species (whole-column heterogeneity) and intraspecimen regions (between-region heterogeneity) were assessed using non-parametric ANOVA (Kruskal–Wallis rank sum test, function kruskal test from R package stats) and post-hoc Dunn test with Holm correction for multiple comparisons (function dunnTest from R package FSA (Ogle and Ogle 2017)).

## Results

### Trabecular bone architecture (TBA)

**Fig. 3:**
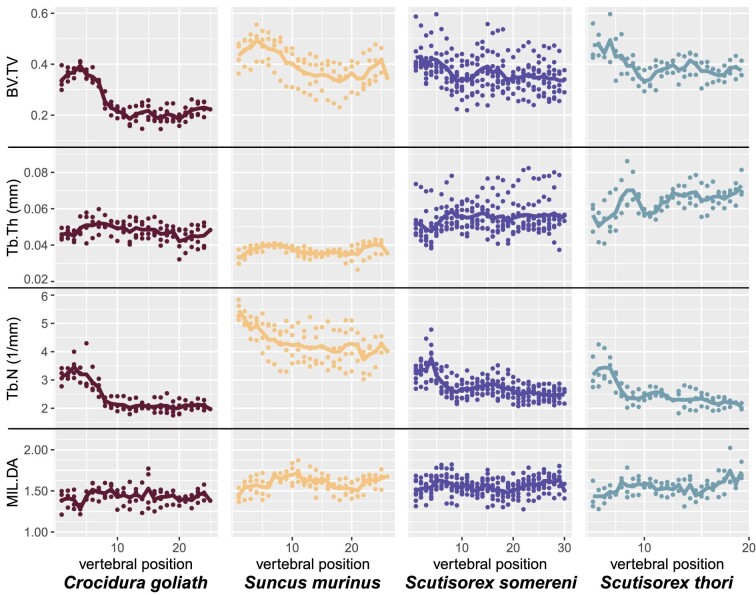
Trabecular bone architecture (TBA) metrics for the entire length of the vertebral column, for all specimens. Values shown (points) are averages of values for cranial and caudal VOIs, but overall trends are the same in plots of only cranial or only caudal VOI values (Figs S5–6). Lines show the average at each vertebral position for a given species.

BV.TV and Tb.N consistently show a clear decrease from the classically defined cervical region (C03–C07; position 1–5) and more cranial thoracics (T01–T05; positions 5–10) to the more caudal thoracics and lumbar regions (positions 10–30; Fig. 3). Tb.Th and MIL.DA stay relatively constant throughout the entire column, although MIL.DA has some sharp local fluctuations, as does Tb.Th in the more cranial positions of *S. thori* (∼C07–T10, position 5–15).

All four species have similar, overlapping ranges for MIL.DA, with *C. goliath* usually representing the lower extremes of the range. BV.TV is also the lowest through the whole column in *C. goliath*, with *S. murinus* and both species of *Scutisorex* having similar ranges ∼15–20% higher than *C. goliath* in the more caudal thoracic and lumbar regions (beginning ∼T05, position 10). *C. goliath* mostly has the lowest values for Tb.N, and although *S. somereni* Tb.N is higher than that of *S. thori*, both are much lower than *S. murinus*, which has 1.5–2 more trabeculae per mm than any other species throughout the column. In contrast, *S. murinus* has the lowest Tb.Th of any species. *Scutisorex* has the highest values for Tb.Th, with *S. thori* having higher average Tb.Th than *S. somereni.*

Results shown in Fig. 3 represent averages of TBA values for cranial and caudal VOIs. Plots of cranial VOI only and caudal VOI only show the same broad patterns, within and across species (Figs. S5–6). However, there are significant differences between cranial and caudal VOI values for all four TBA metrics shown (paired Wilcoxon signed-rank test, *P* < 2.2e-16; Fig. S7). For all taxa, the caudal VOI more frequently has higher values for BV.TV, Tb.N, and MIL.DA; the cranial VOI more frequently has higher values for Tb.Th (Table S3).

### Regionalization

**Fig. 4:**
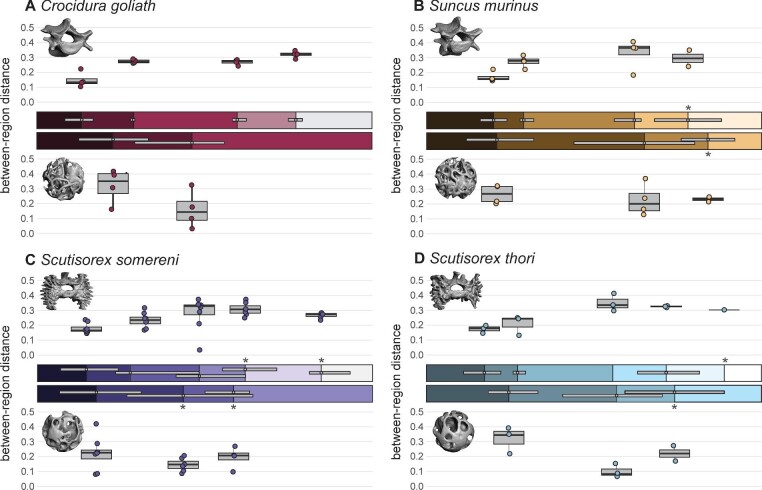
Average number and location of region breaks for gross-morphology and TBA regionalization analyses. In each quadrant (A–D), the top half of the plot shows gross-morphology results, and the bottom half shows TBA results, as indicated by 3D models of whole vertebrae (top) and trabecular bone VOIs (bottom). Each bar shows the average locations of region breaks across all specimens for a species, with different box colors representing different regions. Because there was intraspecific variation in total number of vertebrae across specimens, all breaks are shown as a percentage of the total length of the vertebral column. Horizontal grey bars within regionalization bars show the intraspecific range of locations of a given region break. Boxplots located directly above or below each bar show the between-region heterogeneity of the two adjacent regions. For example, in A, the far-left box on the top plot shows the between-region heterogeneity for the first and second gross morphology regions in *C. goliath*. Points on boxplots represent individual specimens. Asterisks denote region breaks that were not detected in all specimens; for example, a fifth gross-morphology region was only detected in two of four specimens of *S. murinus*. This is also reflected in the boxplot associated with that region break, showing only the values for the two relevant specimens.

Both TBA and external morphology analyses yielded detectable regions using the segmented regression method. However, the detected region breaks do not match between the two analyses (Fig. 4). Analysis of external morphology detected more regions than TBA analysis. Most specimens have five or six external regions, with three exceptions: two specimens of *S. murinus* and one *S. somereni* specimen have four external regions (Figs. S9–10, Table 2). The majority of *Scutisorex* specimens (7 of 11) have six external regions, whereas the maximum number of regions detected in non-*Scutisorex* specimens is five. Most specimens have three or four TBA regions, with a single exception: one specimen of *S. somereni* has two TBA regions (Fig. S10, Table 3).

**Table 2:** Model choices and region breaks for external morphology regionalization analyses. Models are listed as number of regions detected, followed by the Akaike weight for the model in brackets. Region breaks are only listed for the best model as determined by AIC. Breaks occur following the position listed; For example, in FMNH 162144, the first region includes vertebrae 1–4. Asterisks denote specimens in which the Akaike weight is less than 0.8 for the best model.

Specimen number	Taxon	Avg. regions	best model (Akaike wt.)	Total vert	best model region breaks					2^nd^ model (Akaike wt.)	3^rd^ model (Akaike wt.)
162144	*Crocidura goliath*	4.959	5 (0.959)	24	4	7	15	19	–	4 (0.041)	3 (0.000)
162185	*Crocidura goliath*	4.852	5 (0.853)	24	3	7	14	18	–	4 (0.147)	3 (0.001)
162186	*Crocidura goliath*	4.278	5 (0.463)*	24	2	7	14	19	–	4 (0.352)	3 (0.185)
167691	*Crocidura goliath*	4.946	5 (0.946)	25	4	7	15	19	–	4 (0.054)	3 (0.000)
213932	*Suncus murinus*	4.960	5 (0.960)	25	4	7	16	22	–	4 (0.040)	3 (0.000)
213935	*Suncus murinus*	4.133	4 (0.867)	25	6	8	16	–	–	5 (0.133)	3 (0.000)
213944	*Suncus murinus*	4.566	5 (0.648)*	25	5	7	14	17	–	4 (0.269)	3 (0.083)
213945	*Suncus murinus*	4.158	4 (0.842)	25	5	7	16	–	–	5 (0.158)	3 (0.000)
137613	*Scutisorex somereni*	5.547	6 (0.586)*	29	2	7	13	17	21	5 (0.372)	4 (0.041)
148270	*Scutisorex somereni*	5.985	6 (0.985)	30	4	7	14	20	27	5 (0.015)	7 (0.000)
148271	*Scutisorex somereni*	6.168	6 (0.832)	30	3	7	14	19	28	7 (0.168)	5 (0.000)
148941	*Scutisorex somereni*	6.001	6 (0.999)	30	4	7	14	18	23	7 (0.001)	5 (0.000)
160178	*Scutisorex somereni*	5.999	6 (0.998)	29	4	7	13	17	27	5 (0.002)	7 (0.000)
160180	*Scutisorex somereni*	5.569	6 (0.605)*	29	4	7	12	16	24	5 (0.217)	4 (0.131)
223983	*Scutisorex somereni*	5.008	5 (0.992)	28	6	9	15	20	–	6 (0.008)	7 (0.000)
227556	*Scutisorex somereni*	4.049	4 (0.931)	29	7	14	18	–	–	5 (0.059)	3 (0.010)
219669	*Scutisorex thori*	5.999	6 (0.999)	27	5	7	15	17	24	5 (0.000)	4 (0.000)
222612	*Scutisorex thori*	5.011	5 (0.989)	27	3	7	15	22	–	6 (0.011)	4 (0.000)
222613	*Scutisorex thori*	4.991	5 (0.991)	27	6	8	15	19	–	4 (0.009)	6 (0.000)

**Table 3:** Model choices and region breaks for trabecular bone analyses. Models are listed as number of regions detected, followed by the Akaike weight for the model in brackets. Region breaks are only listed for the best model as determined by AIC. Breaks occur following the position listed; For example, in FMNH 162144, the first region includes vertebrae 1–8. Asterisks denote specimens in which the Akaike weight is less than 0.8 for the best model.

Specimen number	Taxon	Avg. regions	best model (Akaike wt.)	Total vert.	best model region breaks			2^nd^ model (Akaike wt.)	3^rd^ model (Akaike wt.)
162144	*Crocidura goliath*	3.002	3 (0.927)	24	8	12	-	4 (0.037)	2 (0.035)
162185	*Crocidura goliath*	2.700	3 (0.687)*	24	7	12	-	2 (0.306)	4 (0.007)
162186	*Crocidura goliath*	3.137	3 (0.685)*	24	3	7	-	4 (0.221)	2 (0.091)
167691	*Crocidura goliath*	3.001	3 (0.999)	25	4	14	-	4 (0.001)	2 (0.000)
213932	*Suncus murinus*	3.157	3 (0.843)	25	8	20	-	4 (0.157)	5 (0.000)
213935	*Suncus murinus*	3.121	3 (0.879)	25	4	16	-	4 (0.121)	5 (0.000)
213944	*Suncus murinus*	3.834	4 (0.727)*	25	6	18	23	3 (0.220)	5 (0.053)
213945	*Suncus murinus*	3.968	4 (0.967)	25	3	11	19	3 (0.033)	5 (0.000)
137613	*Scutisorex somereni*	3.117	3 (0.874)	29	4	14	-	4 (0.119)	2 (0.005)
148270	*Scutisorex somereni*	2.973	3 (0.967)	30	7	17	-	2 (0.030)	4 (0.003)
148271	*Scutisorex somereni*	3.716	4 (0.826)	30	3	13	19	2 (0.110)	3 (0.064)
148941	*Scutisorex somereni*	4.063	4 (0.937)	30	2	8	15	5 (0.063)	3 (0.000)
160178	*Scutisorex somereni*	2.462	2 (0.491)*	29	9	-	-	3 (0.461)	1 (0.031)
160180	*Scutisorex somereni*	4.051	4 (0.953)	29	3	8	17	5 (0.043)	6 (0.004)
223983	*Scutisorex somereni*	3.039	3 (0.934)	28	8	18	-	4 (0.053)	2 (0.013)
227556	*Scutisorex somereni*	3.216	4 (0.449)*	29	5	11	18	3 (0.318)	2 (0.233)
219669	*Scutisorex thori*	3.615	4 (0.610)*	27	9	16	24	3 (0.375)	2 (0.008)
222612	*Scutisorex thori*	4.036	4 (0.964)	27	6	11	16	5 (0.036)	3 (0.000)
222613	*Scutisorex thori*	2.987	3 (0.864)	27	5	19	-	2 (0.075)	4 (0.061)

Locations of some analytically-derived external region breaks in the vertebral series are similar to classically-defined region breaks. Like most mammals, all specimens in the sample have seven cervical positions, with the last cervical at position 5 as numbered here. In our analyses of external morphology, we recovered a region break at position 4–6 in 13 of 19 specimens, indicating detection of the cervical-thoracic transition (Table 2). In *Crocidura* and *Suncus*, the classically defined thoracolumbar break occurs after position 19 or 20, and in *Scutisorex* it occurs after position 18 or 19 (Table 1). Overall, we detected an external region break at position 17–20 in 13 of 19 specimens (not precisely the same set of specimens as with a break in positions 4–6). The location of this region break also roughly corresponds to the diaphragmatic vertebra or “Diaphragmatic Joint Complex” (Filler 2007) (Table 1), which is transitional between sections of the thoracolumbar column that restrict (prediaphragmatic) and allow (postdiaphragmatic) flexion in the sagittal plane (Slijper 1946; Filler 2007; Randau and Goswami 2017).

In addition to the external region breaks that approximate classically-defined vertebral regions, we recovered several additional breaks. Two of these are similar to regions detected by Jones et al. 2018: a break at position 7–9 (17 of 19 specimens), corresponding to the caudal end of the “pectoral” region of Jones et al. 2018; and a break at position 14–16 (14 of 19 specimens), corresponding to the cranial end of their “posterior dorsal” region (Table 2). The third additional external region break we detected is present only in species of *Scutisorex*, and occurs between positions 21 and 28 (8 of 11 *Scutisorex* specimens, including only 1 of 3 *S. thori specimens*), placing it in the mid-to-caudal lumbar region (Table 2).

Region break locations in the TBA segmented regression analyses are less consistent than those of the external morphology analyses, but some patterns do emerge. All specimens have at least one TBA region break among positions 2–9; three specimens have two region breaks in that span (one at 2 or 3 and one at 7 or 8; Table 3). Eight specimens have a break at position 11–14, and 12 specimens have a break among positions 15–19, a span encompassing all observed locations of the diaphragmatic vertebra. Only three specimens have TBA region breaks caudal to position 19: two *S. murinus* (position 20 and 23) and one *S. thori* (position 24).

Support for the most likely configuration of region breaks is mostly high, above 0.80. Mean support for the chosen model is slightly higher in external morphology analyses (mean Akaike weight 0.87; Table 2) than in TBA analyses (mean Akaike weight = 0.82; Table 3). Perhaps the clearest outlier in this regard is the chosen TBA region model for FMNH 160178 (*S. somereni*), a 2-region model with an Akaike weight of 0.49. The Akaike weight for the second most likely model for this specimen (a 3-region model) is only slightly lower (0.46). Although one other specimen of *S. somereni* (FMNH 227556) also has a very low Akaike weight for its best model (4 regions, Akaike weight = 0.45), the weight for its second most likely model is much lower (0.32).

### Heterogeneity

**Fig. 5:**
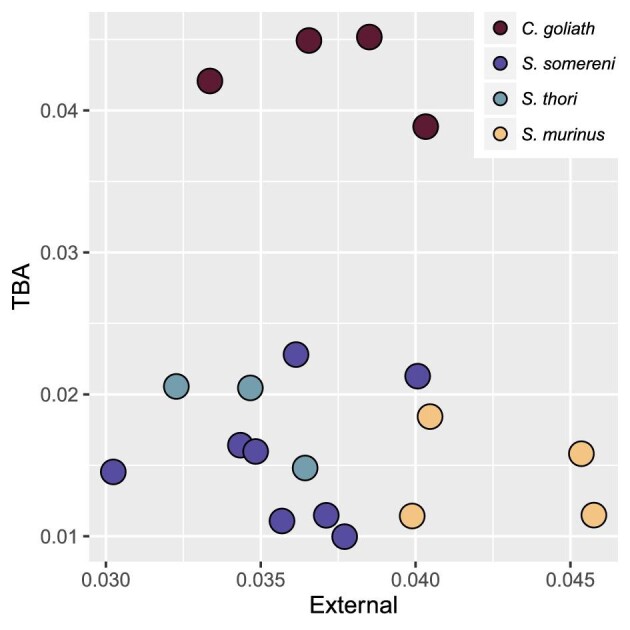
Scatterplot of whole-column heterogeneity for external morphology (y-axis) and TBA (x-axis). The method of calculating heterogeneity follows that of Jones et al. (Jones et al. 2018) (see Methods, Whole-column and between-region heterogeneity). Pearson correlation = -0.09, *P* = 0.7.

*Suncus murinus* has significantly higher external morphology whole-column heterogeneity than that of the other three species (*P* ≤ 0.1, Table 4, Figs. 5, S12), although the magnitude of difference among species is relatively low. In contrast, *C. goliath* has the highest whole-column heterogeneity in TBA, and the magnitude of difference between heterogeneity ranges of *C. goliath* and the other three species is relatively large. Only *S. somereni* and *S. murinus* were recovered as having significantly lower heterogeneity than *C. goliath* (Table 4), although the range of *S. somereni* fully encompasses that of *S. thori* (Figs. 5, S12). There is no correlation between external and TBA whole-column heterogeneity (Pearson correlation = −0.09, *P* = 0.7).

**Table 4:** Results of post-hoc Dunn test, following significant results of non-parametric ANOVA of whole-column heterogeneity across taxa (Kuskal–Wallis; *P* < 0.10). All values are Holm-corrected p-values. Lower left corner shows results for whole-column heterogeneity in gross morphology; upper right triangle shows results for whole-column heterogeneity in TBA. Statistically significant values (*P* < 0.1) in bold.

	*C. goliath*	*S. murinus*	*S. somereni*	*S. thori*
*C. goliath*		**0.035**	**0.025**	0.45
*S. murinus*	**0.10**		0.80	1.00
*S. somereni*	0.47	**0.0090**		0.85
*S. thori*	0.23	**0.0066**	0.48	

For external morphology measurements, between-region heterogeneity was significantly different only when comparing the break between regions 1 and 2 (lower values), and the breaks between regions 3 and 4 or 4 and 5 (higher values; Fig. 4, Table 5). Although non-parametric ANOVA yielded significant results for all four species, the post-hoc Dunn test yielded no significant results for *S. thori*. Yet, *S. thori* was the only species for which comparison of TBA between-region heterogeneity yielded a significant result, with a significantly larger distance between first and second TBA regions than between second and third TBA regions (Table 5).

**Table 5:** Results of non-parametric ANOVAs of intraspecific between-region heterogeneity. Statistically significant values (*P* < 0.1) in bold. Post-hoc Dunn test p-values were calculated using Holm correction for multiple comparisons. Only significant results of post-hoc comparisons are listed, and are notated as follows: “1:2” represents region disparity between regions 1 and 2. “1:2/4:5” represents the post-hoc comparison between magnitude of disparity between regions 1 and 2, and 4 and 5. For *S. thori*, no post-hoc test yielded a significant result at an alpha level of 0.1, despite significant results of the non-parametric ANOVA.

		External morphology	TBA
*C. goliath*	Kruskal–Wallis	*P* = **0.0066**	*P* = 0.14
	Post-hoc (Dunn)	(1:2/4:5) *P* = **0.0030**	—
*S. murinus*	Kruskal–Wallis	*P* = **0.061**	*P* = 0.78
	Post-hoc (Dunn)	(1:2/3:4) *P* = **0.053**	—
*S. somereni*	Kruskal–Wallis	*P* = **0.0012**	*P* = 0.19
	Post-hoc (Dunn)	(1:2/3:4) *P* = **0.0052**	—
		(1:2/4:5) *P* = **0.0024**	
*S. thori*	Kruskal–Wallis	*P* = **0.051**	*P* = **0.062**
	Post-hoc (Dunn)	—	(1:2/2:3) *P* = **0.059**

## Discussion

### Trabecular bone architecture is regionalized along the soricid vertebral column

We investigated regionalization of the soricid vertebral column with two different sets of morphological data: linear measurements of gross morphology and measurements of TBA. Although other studies have reported on quantitative measures of morphological disparity, integration, modularity, and regionalization in vertebral series, this is the first study to investigate morphofunctional regionalization in TBA of mammalian vertebrae. We found evidence of three to four morphologically distinct TBA regions, with regional distinctiveness and functional implications varying throughout the series.

The cranialmost TBA region we detected was the most uniform in length and break point across species (Fig. 4). The vertebrae in this region usually include all cervical positions measured (C03–C07), and often one or two thoracic positions. In a few cases (one *C. goliath*, two *S. somereni*), there are two region breaks in this section, but for most specimens, the “cervical-type” TBA region ends between positions 4 and 9. This region is distinguished by relatively high Tb.N, resulting in high BV.TV (Fig. 3).

A distinct cervical TBA “type” has also been reported in humans, and bears the same hallmarks as we find here: increased BV.TV and increased Tb.N relative to thoracic and lumbar TBA (Acquaah et al. 2015). Additionally, cervical vertebrae exhibit higher bone mineral density than other vertebrae (Yoganandan et al. 2006). From a morphofunctional perspective, high BV.TV is the best structural predictor of high trabecular bone strength, and apparent density (a combination of tissue density and BV.TV) is the best predictor of trabecular bone material properties such as high elastic modulus and high failure strength (Keaveny et al. 2001; Keller et al. 2001; Guo and Kim 2002; Nazarian et al. 2008; Oftadeh et al. 2015; Musy et al. 2017). Other work suggests that high BV.TV is also related to a greater *variety* of forces acting on the bone, causing the trabecular structure to be remodeled with additional cross-struts and decreased anisotropy (Smit et al. 1997). Combined, this evidence suggests that the cervical region, or, more specifically, the “cervical-type” vertebral trabecular bone, is adapted to withstand a diverse set of forces and have strong failure resistance.

Jones et al. 2020 showed that the cervical and anterior thoracic regions have higher range of motion (ROM) than most other regions when considering dorsoventral and lateral flexion. A similar pattern was reported in artiodactyls (Belyaev et al. 2021), where the peak bending ROM was at approximately C05 (position 4 in their analyses). This roughly corresponds to the peak of BV.TV and Tb.N in the shrew columns we studied, which occurs at C05 or C06 (Fig. 3). It is possible, then, that the range of motion in the mammalian neck is positively correlated with the BV.TV and Tb.N of the vertebral centra in a consistent way. Correspondence between these two signals should be further investigated using both ROM and TBA data from the same specimens (see below, Mapping Functional Regionalization).

More caudal TBA regions have less distinct morphological characteristics than the cervical-type region, and have less consistent boundaries across taxa (Figs. 3 and 4, Table 3). There is a somewhat consistent region break near the diaphragmatic vertebra (Table 3), but any broader-scale pattern of TBA characteristics correlated with this region break is obscure (Fig. 3). We might expect this pattern to be reflected by between-region heterogeneity, with the difference between TBA region 1 and other TBA regions being much higher than among regions 2, 3, and 4. We found that region 1:2 heterogeneity is, on average, slightly higher than region 2:3 heterogeneity (Fig. 4), but the difference is not significant except in *S. thori* (Table 5).

### Inexact match between gross morphology regions and TBA regions

TBA regions do not match gross morphology regions, and are less consistent in location and size within and across taxa (Fig. 4, S8–11). To explore this result, we considered the variety of developmental and functional controls on vertebral morphology across scales. Morphogenesis of the vertebrate axial column is controlled by *Hox* genes (Wellik 2007; Wellik 2009; Mallo et al. 2010; Böhmer 2017), with overlapping areas of influence from various *Hox* paralogs producing particular characteristics in vertebrae (e.g., Mallo et al. 2010). We therefore assume that there is some developmental and phylogenetic signature in the gross morphology of mammalian vertebrae (e.g., Asher et al. 2011; Jones 2015; Kivell 2016; Martín-Serra et al. 2021; Figueirido et al. 2021). It is less clear what degree of influence phylogeny and development have on adult vertebral TBA.

The principles of bone functional adaptation (Wolff 1893; Cowin 1986; Ruff et al. 2006) indicate that bone is remodeled to best handle in-vivo force regimes. Trabecular bone experiences faster turnover than cortical bone (Manolagas 2000), and might therefore be expected to be more thoroughly overprinted with functional signals than gross bone morphology. Studies of human vertebral development have shown ontogenetic increases in BV.TV of fetuses, raising the possibility that fetal movement might cause functional overprinting even before birth (Skerry 2000). Yet, human fetal increases in BV.TV occur without a correlated increase in anisotropy, and occur throughout gestation, even when fetal movement becomes more limited closer to term (Acquaah et al. 2015). The lack of increasing anisotropy, which we would expect under bone functional adaptation, suggests that trabecular bone is developmentally programmed to have specific characteristics at birth. Those characteristics change rapidly when force is applied to bones postpartum: BV.TV drops and DA increases (Tanck et al. 2001; Acquaah et al. 2015), and the angle between primary load direction and direction of the greatest bone stiffness decreases (Tanck et al. 2001).

It is therefore not surprising that TBA regionalization patterns do not match those of gross morphology. If gross morphology and at-birth TBA are correlated in any way due to developmental or phylogenetic influences, much of that correlation could quickly be obscured by functional overprinting of the trabecular bone (but see above regarding phylogenetic differences in TBA across adult shrews). Overprinting also may help to explain the variability of TBA region break locations relative to the consistency of gross morphology regions. If forces are the primary determinant of TBA, their signals may be spread out across the many closely-interacting subunits of the vertebral column. This may result in more gradual transitions between functional regions than between morphological regions, because morphological regions are subject to abrupt changes in the morphogenetic control of particular *Hox* genes. To add an additional layer of complexity, intraspecific variation in behavior and associated mechanical environment may have an effect on where vertebral TBA regions manifest. However, research on the link between behavior and trabecular bone remodeling typically consists of coarse experimental manipulations (e.g., Barak et al. 2011), not the more subtle behavioral differences expected in a wild setting.

### Mapping functional regionalization across morphological scales

Our results agree with those of Jones et al. 2020 because regions derived from functional traits (in their case, ROM; in our case, TBA) do not match regions derived from external vertebral morphology. Jones et al. 2020 also found that functional regions often span more than one morphological region, and that the functional diversity of the mammalian spine likely originated from derivation of new functional capabilities from preexisting morphological diversity (Jones et al. 2020). This is an interesting result in the context of our study because we uncovered an extra gross morphology region in both species of *Scutisorex* relative to *S. murinus* and *C. goliath*, and relative to mammals more broadly (Jones et al. 2018). This new *Scutisorex* region break occurs in the mid-to-caudal end of the lumbar spine, and does not relate to any TBA region we recovered (Fig. 4, Table 2). If we interpret this through the lens of the findings of Jones et al. 2020, we might infer that this region does not have a distinct function, but could be an example of the latent morphological diversity that precedes functional diversity. However, we suggest that it is more likely a sign that TBA and ROM do not detect the same morphofunctional signals. Though the two sets of signals might occasionally be aligned (cervical spine discussed above), they capture different functional aspects of the same structure at different scales.

The reason for this interpretation is that there is evidence of an additional functional region in the caudal lumbar positions of *Scutisorex*, based on current and previous morphological examinations. There is a precipitous drop in the number of accessory articulations (tubercles) present on the slablike transverse processes in the last few vertebral positions of both species of *Scutisorex* (Smith and Angielczyk 2020). Because the tubercles act to restrict motion in several directions (Cullinane et al. 1998; Cullinane and Bertram 2000), it is logical to predict that the loss of tubercles would relate to a change in ROM, and potentially an additional functional region. It is easy to speculate on why this additional region with higher ROM would be advantageous for *Scutisorex*: the motion of its lumbar spine is restricted to primarily sagittal flexion (Cullinane et al. 1998), and a more mobile region caudal to this extremely stiff structure would permit it to move less awkwardly in certain situations (e.g., turning a tight corner, which is difficult with a spine that restricts lateral bending (Cullinane et al. 1998)). As such, despite the lack of a caudal-lumbar TBA region, *Scutisorex* may still have a distinct functional region in the lumbosacral part of the spine, but our chosen functional correlate was not an appropriate choice to detect it.

### Consideration of ecological signal

Unlike the cross-species similarity of the cervical-type region, the TBA regions surrounding the diaphragmatic vertebra show species-specific differences (Figs. 4, S8–11). Although *Scutisorex* is likely semifossorial (Churchfield et al. 2007), and *C. goliath* and *S. murinus* are terrestrial/generalized (Wilson and Reeder 2005; Jacquet et al. 2013; Jacquet et al. 2015), we were unable to identify corresponding ecological signals in TBA region characteristics. It is true that the TBA region breaks in *S. thori* and *S. somereni* are similar (Figs. 4, S10–11), but both also resemble the TBA region patterning of *S. murinus* (Table 3), with detection of a fourth TBA region in about 50% of specimens in both genera. This is in contrast to *C. goliath*, which never shows a fourth TBA region, and has its final TBA region break at a more cranial position than the other species (Fig. 4, S8).

One potential ecological signal we examined was related to axial torsion during forelimb digging. Because axial torsion ROM in typical mammalian spines is highest in the thoracic region (Jones et al. 2018; Jones et al. 2020; Belyaev et al. 2021), it is possible that forelimb diggers would rotate the pectoral girdle and forelimbs axially while digging the sides of tunnels (Belyaev et al. 2021). Axial rotation is probably even more localized in *Scutisorex*, due to the accessory articulations (tubercles) in the caudal thoracic region that arrest axial rotation (Cullinane et al. 1998; Cullinane and Bertram 2000; Smith and Angielczyk 2020). We suspect that such a change in the direction of force transmission through the thoracic column might result in a portion of the thoracolumbar column with an increased capability to withstand forces in multiple directions, evidenced by an increase in Tb.N and BV.TV, and a decrease in DA. There is a very slight increase in BV.TV between positions 10 and 20 in the columns of both *Scutisorex* species, but it is not dramatic (Fig. 3); Tb.N and DA remain fairly stable through this section. Neither *Scutisorex* species shows an appreciable change relative to the two terrestrial species in any metric that correlates with the axial rotation-digging hypothesis, although digging behavior in general may have some effect on vertebral TBA (Zack et al. 2021; Amson and Bibi 2021), especially in the lumbar spine, which is stiffened in digging taxa (Gaudin and Biewener 1992; Gaudin 2011; Jones et al. 2018).

We also assessed potential signals of higher-stress force regimes in the lumbar spines of *Scutisorex*. In both species, the last TBA region break usually occurs within a few positions of the first lumbar. We interpret this caudalmost TBA region as a functional region encompassing the most modified section of the vertebral column. If this region is specifically adapted to withstand strong forces in the axial direction while craniocaudally compressed to form a rigid beam (Cullinane and Bertram 2000; Smith and Angielczyk 2020), we might expect a high degree of anisotropy in the axial direction, and higher trabecular thickness (Keaveny and Hayes 1993). There is an increase in DA in both species between position 20 and 30 (which usually covers most or all of the caudalmost detected TBA region), and a slight increase in Tb.Th in *S. thori* in the same region. However, this slight increase in DA is also present in the final six to seven positions of *S. murinus*, so it may be a signal of a “lumbar-type” TBA rather than a signal specific to the enigmatic use of the *Scutisorex* spine.

It is important to note that even though *S. murinus* and *C. goliath* were chosen as representatives of “generalized” shrew morphology and ecology, their trabecular bone morphology is strikingly different, and it is unclear which is more representative of the “typical” shrew vertebral TBA pattern. Through the whole column, *S. murinus* has high BV.TV by way of high Tb.N, whereas *S. thori* and *S. somereni* have high BV.TV by way of high Tb.Th and moderate Tb.N. *Crocidura goliath* has relatively low BV.TV in comparison, with low-to moderate Tb.Th and low Tb.N. A more phylogenetically broad examination of shrew TBA is needed to understand the degree of variation in vertebral bone characteristics in this large and ecologically diverse group. In fact, the surprising degree of trabecular bone diversity being uncovered across mammals (e.g., Amson and Bibi 2021) is an indicator that much work is needed to understand intra- and inter-specific variation and function of trabecular tissue.

## Conclusions

We found evidence of TBA regionalization along the vertebral column in a group of small mammals (large shrews, family Soricidae, subfamily Crocidurinae). Configuration of TBA regions in the spine does not match that of regions derived from external morphology, or regions derived from other functional characteristics examined in other mammals (Jones et al. 2020), although it is possible that there is some overlap across functional signals of different types (e.g., ROM and trabecular bone adaptation in the cervical series). Future studies should explicitly link ROM and TBA signals within individuals to assess the degree of correlation between them. Although we did not find clear ecological correlates in the TBA of species we examined, we did uncover one additional external morphology region in *Scutisorex*, indicating that the caudalmost portion of the lumbar spine may serve a distinct purpose in the genus relative to other shrews. Additional levels of scale (e.g., whole-column studies and bone crystalline microstructure) may give further insights into vertebral function, not only in *Scutisorex* and other shrews, but across vertebrates.

Based on our results and those of other recent studies of bone microstructure and vertebral morphology, there is much to be discovered about the morphofunctional diversity of mammalian bone. Further consideration of the relationships among morphogenesis, ontogeny, bone remodeling, function, and locomotor ecology is warranted to build more robust and accurate models of axial column function in vertebrate animals.

## Supplementary Material

obac006_Supplemental_File

## Data Availability

The datasets generated and analysed during the current study are available in the Dryad digital repository, https://doi.org/10.5061/dryad.1g1jwstxc.
