# Supplementary material for: A Shrewd Inspection of Vertebral Regionalization in Large Shrews (Soricidae: Crocidurinae)
Source: Integr Org Biol. 2022 Feb 10;4(1):obac006. doi: 10.1093/iob/obac006 (PMC8915212; doi:10.1093/iob/obac006)
Supplement: obac006_Supplemental_File [file obac006_supplemental_file.pdf]

ELECTRONIC SUPPLEMENTARY MATERIAL

A. SEGMENTATION COMPARISONS AND CHOICE OF DEEP LEARNING MODEL

To assess the effectiveness of our deep learning (DL) neural network segmentation models (classifiers) relative to threshold-based methods, we compared segmentation performance across all specimens using four methods: user-defined grey value thresholding; Otsu thresholding; our DL classifier based on the anterior-column scan of FMNH 162186 (*Crocidura goliath*); and our DL classifier based on the anterior-column scan of FMNH 148271 (*Scutisorex somereni*). See main text for information on classifier training. DL models are available on Dryad (https://doi.org/10.5061/dryad.1g1jwstxc). For each scan included in our dataset, we subjected a slice of a single VOI to all four of these methods. We used the caudal VOI in the vertebral position representing the midpoint of the column section captured in the scan. For example, in a scan of lumbar vertebrae L1-L5, we used the slice at the craniocaudal midpoint of the caudal VOI of L3. This approach generally ensured enough bone present to assess model performance, as caudal VOIs tended to be larger due to centrum morphology.

As shown below in Figure S1-S4, the segmentation accuracy of the DL classifiers equaled or exceeded threshold-based techniques. Here we defined accuracy as the capability to capture all bone pixels within the segment without capturing background pixels. We included the threshold-based methods in these figures for comparative purposes only, as they were not used for any segmentation in the final dataset. They serve to demonstrate the utility of DL classifiers for our specific segmentation needs. For example, in the anterior-column scan of *C. goliath* FMNH 162185 (Figure S1), both DL classifiers were able to detect an area of background pixels on viewer left of the slice corresponding to a small hole in the bony structure. This was not detected by either of the threshold-based methods, likely because a threshold value high enough to detect such a hole would exclude too many other true bone pixels from the segment. Not all scans showed a high degree of variation in performance among the four methods (e.g., lumbar-column scan of *C. goliath* FMNH 167691, Figure S1). In the case of an apparent tie in classifier performance, results from both DL classifiers were carefully inspected across multiple slices to determine a winner. Table S1 also lists chosen classifier, as well as scan resolution.

FIGURE S1, comparison of four segmentation methods across all included scans of *Crocidura goliath*. All slices shown are from the craniocaudal midpoint of the caudal VOI of the listed vertebral position. Far left column shows original unsegmented grey value image. Asterisks denote the chosen DL classifier for a given specimen.

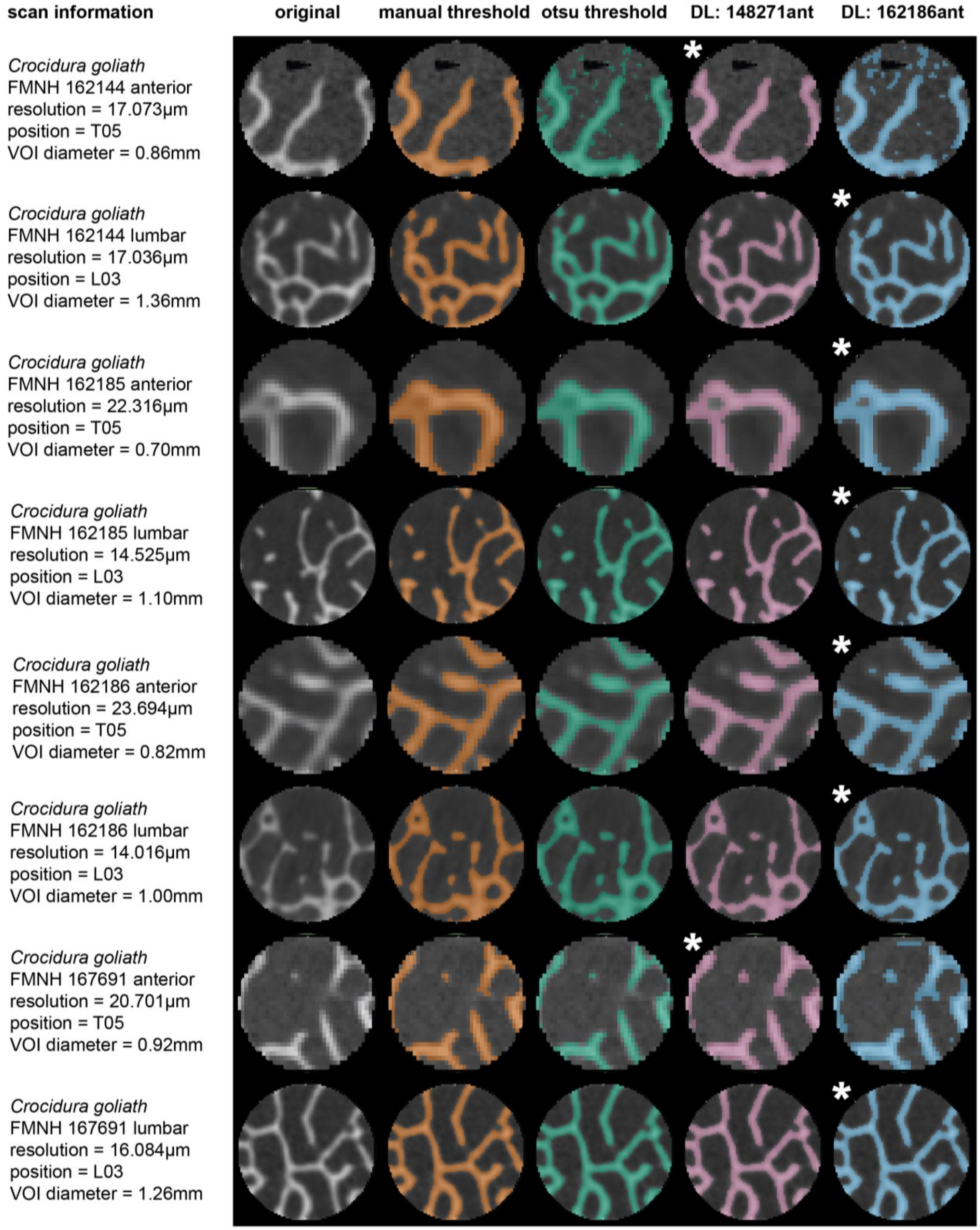

FIGURE S2, comparison of four segmentation methods across all included scans of *Suncus murinus*. All slices shown are from the craniocaudal midpoint of the caudal VOI of the listed vertebral position. Far left column shows original unsegmented grey value image. Asterisks denote the chosen DL classifier for a given specimen.

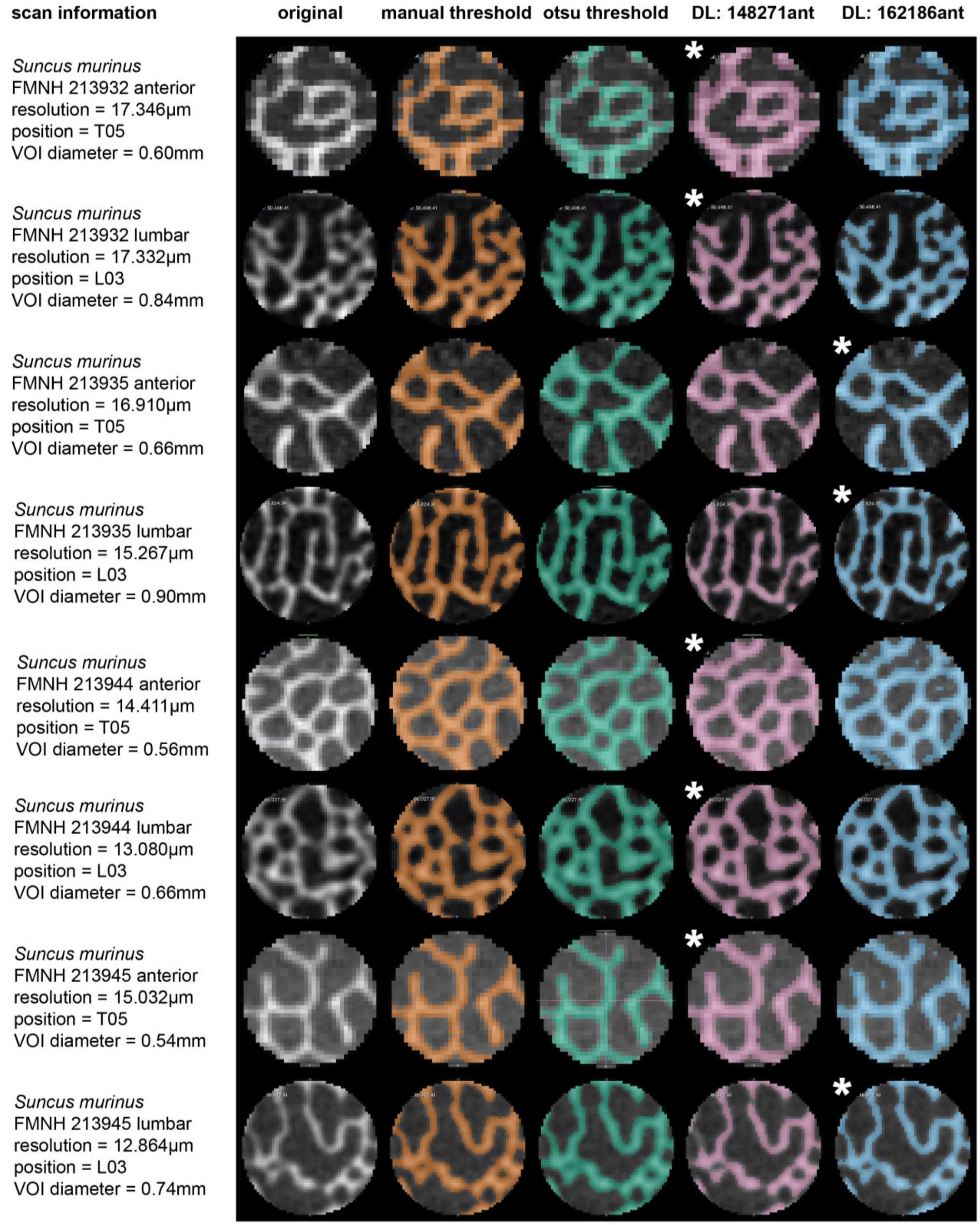

FIGURE S3, comparison of four segmentation methods across all included scans of *Scutisorex somereni*. All slices shown are from the craniocaudal midpoint of the caudal VOI of the listed vertebral position. Far left column shows original unsegmented grey value image. Asterisks denote the chosen DL classifier for a given specimen.

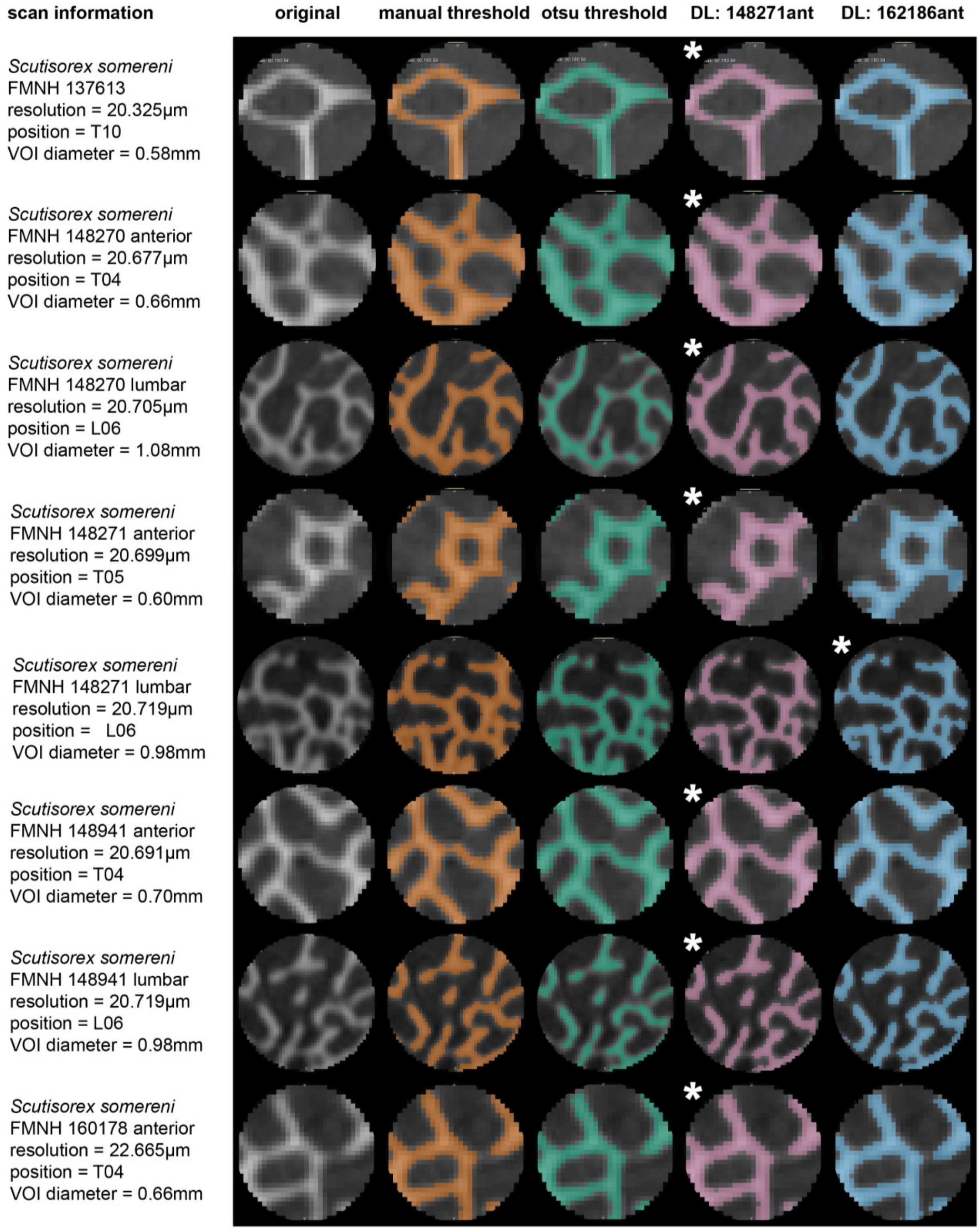

FIGURE S3, continued.

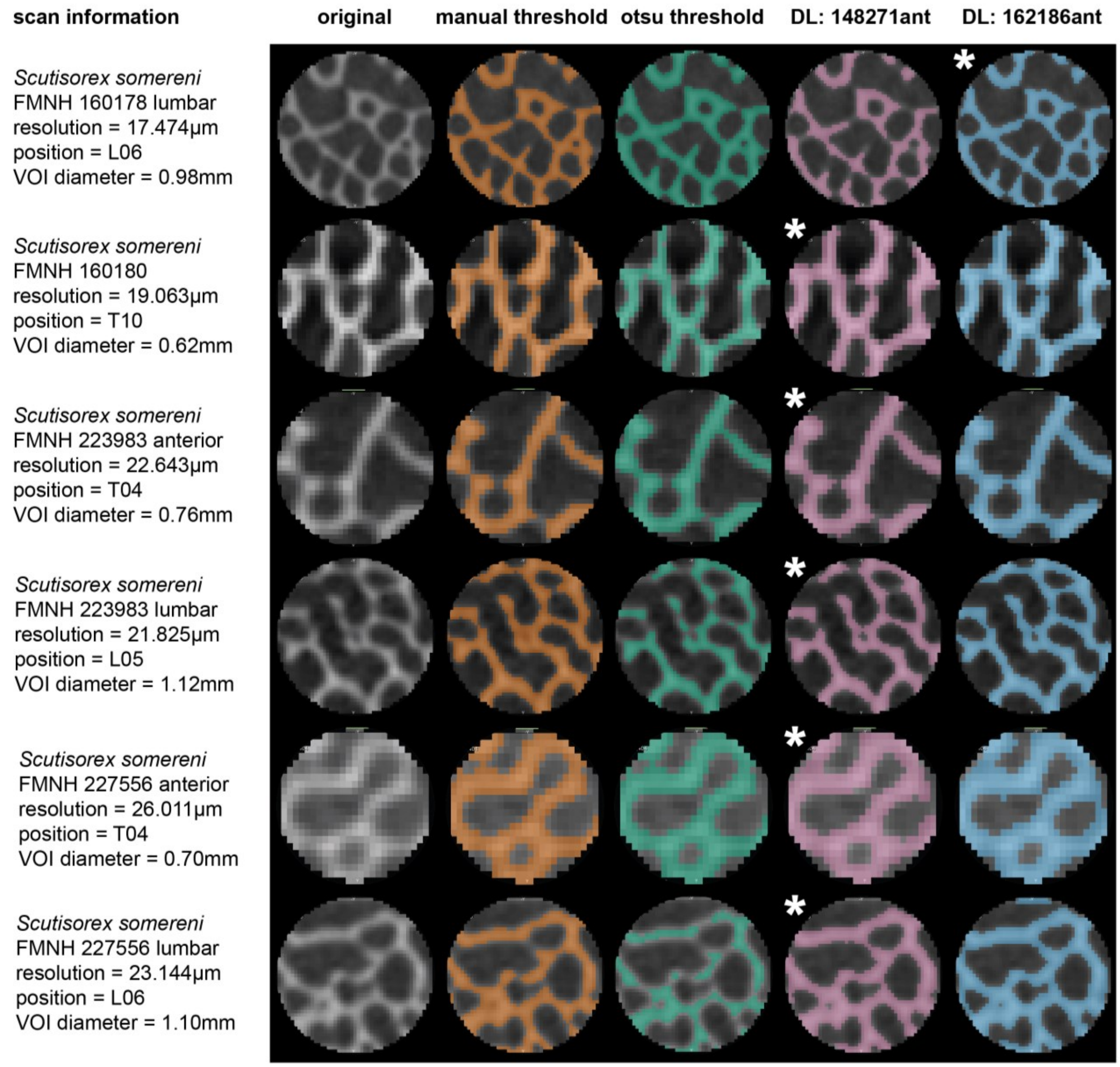

FIGURE S4, comparison of four segmentation methods across all included scans of *Scutisorex thori*. All slices shown are from the craniocaudal midpoint of the caudal VOI of the listed vertebral position. Far left column shows original unsegmented grey value image. Asterisks denote the chosen DL classifier for a given specimen.

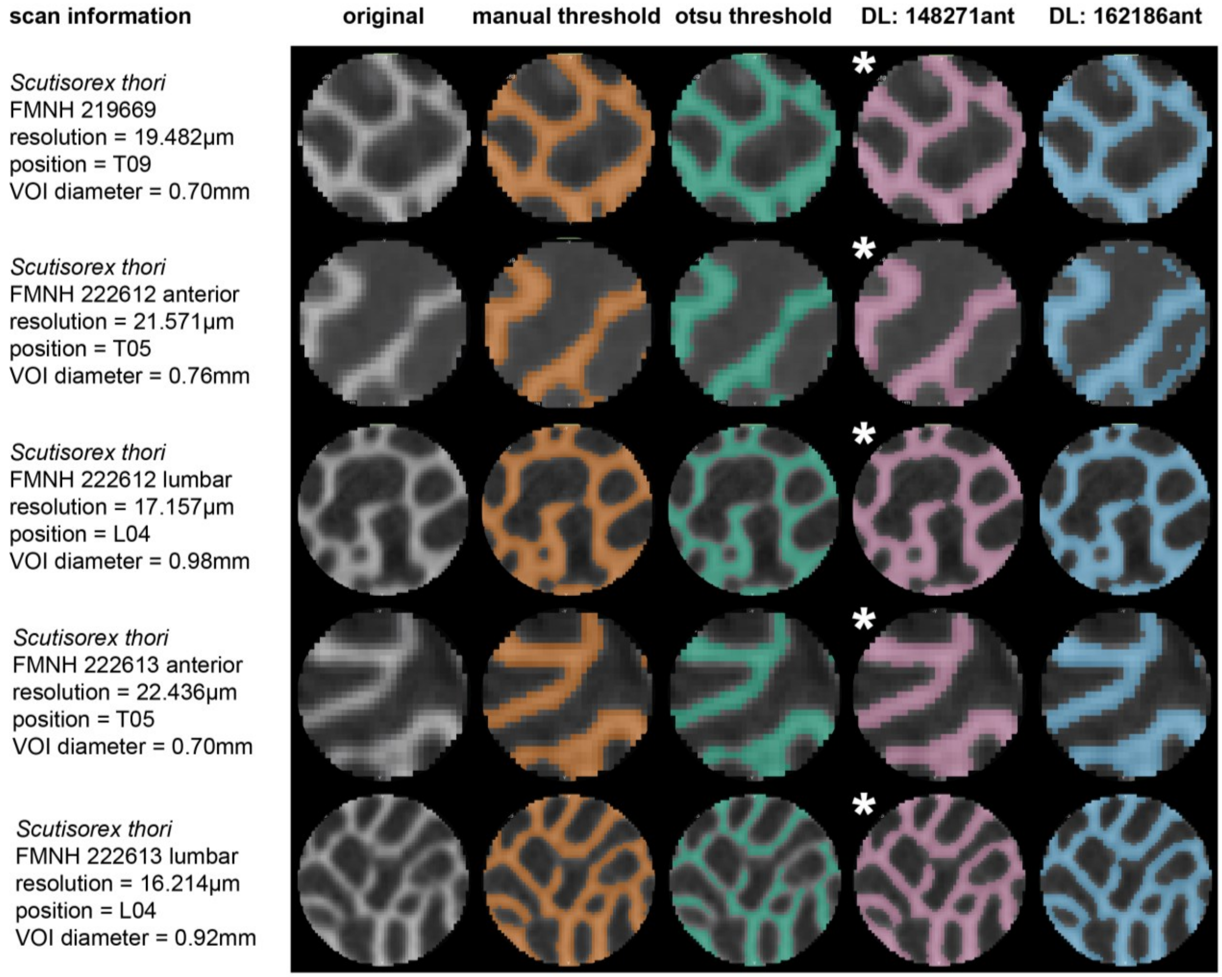

TABLE S1, μCT scan resolution for all component scans of all specimens in our study, and the Deep Learning model chosen to segment each. Under Scan coverage, C, T, and L refer to cervical, thoracic, and lumbar regions, respectively. For details on model choice, see Figure S1-S4.

Specimen No.	Taxon	Scan coverage	Scan resolution (μm)	Segmentation model
FMNH 162144	*Crocidura goliath*	C/T	17.073	148271
		L	17.036	162186
FMNH 162185	*Crocidura goliath*	C/T	22.316	162186
		L	14.525	162186
FMNH 162186	*Crocidura goliath*	C/T	23.694	162186
		L	14.016	162186
FMNH 167691	*Crocidura goliath*	C/T	20.701	148271
		L	16.084	162186
FMNH 213932	*Suncus murinus*	C/T	17.346	148271
		L	17.332	148271
FMNH 213935	*Suncus murinus*	C/T	16.910	162186
		L	15.267	162186
FMNH 213944	*Suncus murinus*	C/T	14.411	148271
		L	13.080	148271
FMNH 213945	*Suncus murinus*	C/T	15.032	148271
		L	12.864	162186
FMNH 137613	*Scutisorex somereni*	C/T/L	20.325	148271
FMNH 148270	*Scutisorex somereni*	C/T	20.677	148271
		L	20.705	148271
FMNH 148271	*Scutisorex somereni*	C/T	20.699	148271
		L	20.719	162186
FMNH 148941	*Scutisorex somereni*	C/T	20.691	148271
		L	20.719	148271
FMNH 160178	*Scutisorex somereni*	C/T	22.665	148271
		L	17.474	162186
FMNH 160180	*Scutisorex somereni*	C/T/L	19.063	148271
FMNH 223983	*Scutisorex somereni*	C/T	22.643	148271
		L	21.825	148271
FMNH 227556	*Scutisorex somereni*	C/T	26.011	148271
		L	23.144	148271
FMNH 219669	*Scutisorex thori*	C/T/L	19.482	148271
FMNH 222612	*Scutisorex thori*	C/T	21.571	148271
		L	17.157	148271
FMNH 222613	*Scutisorex thori*	C/T	22.436	148271
		L	16.214	148271

B. ADDITIONAL SUPPLEMENTARY FIGURES

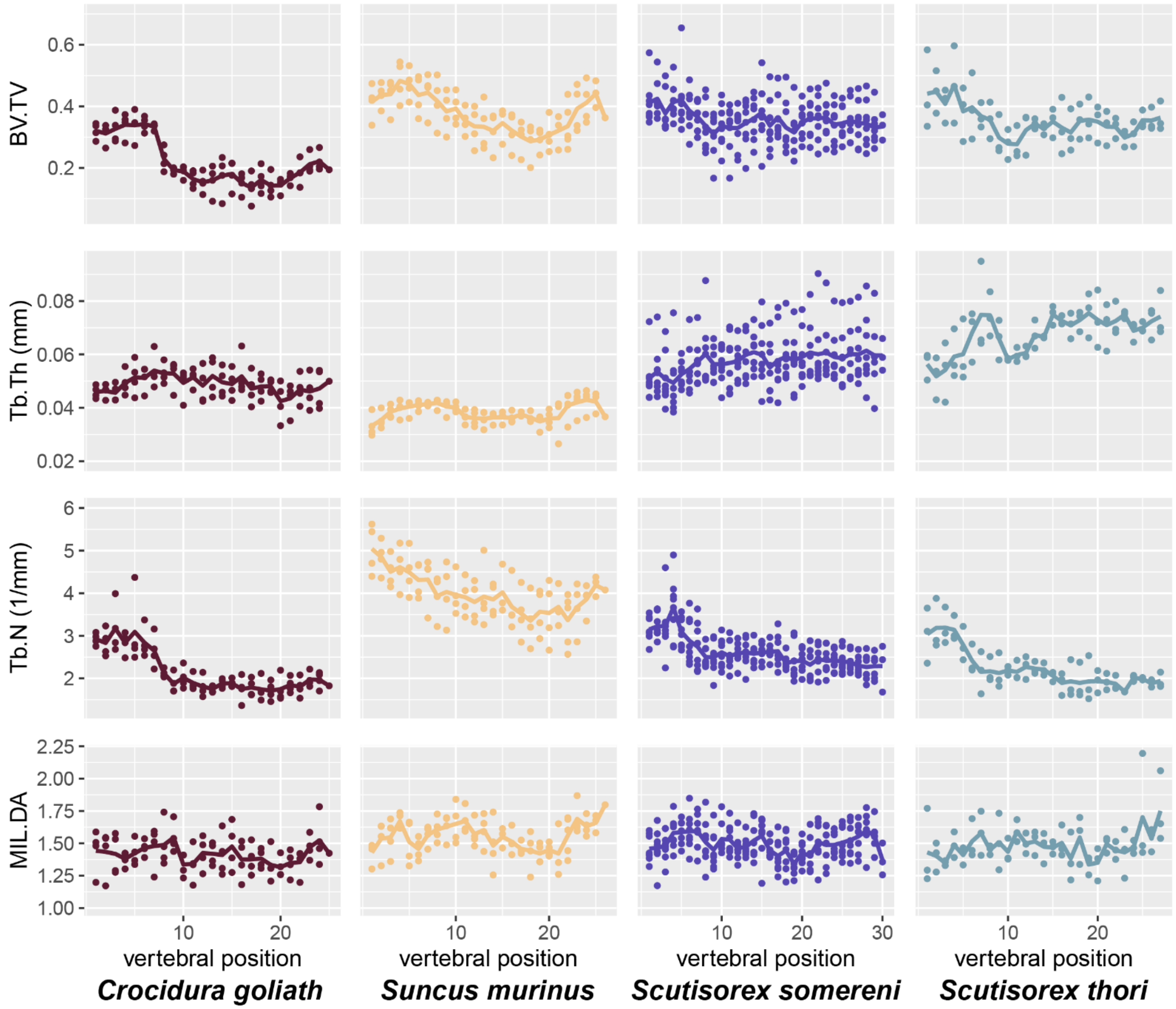

FIGURE S5, Trabecular bone architecture (TBA) metrics for cranial VOIs only. Each point represents the value for the cranial VOI of a single specimen at a given vertebral position.

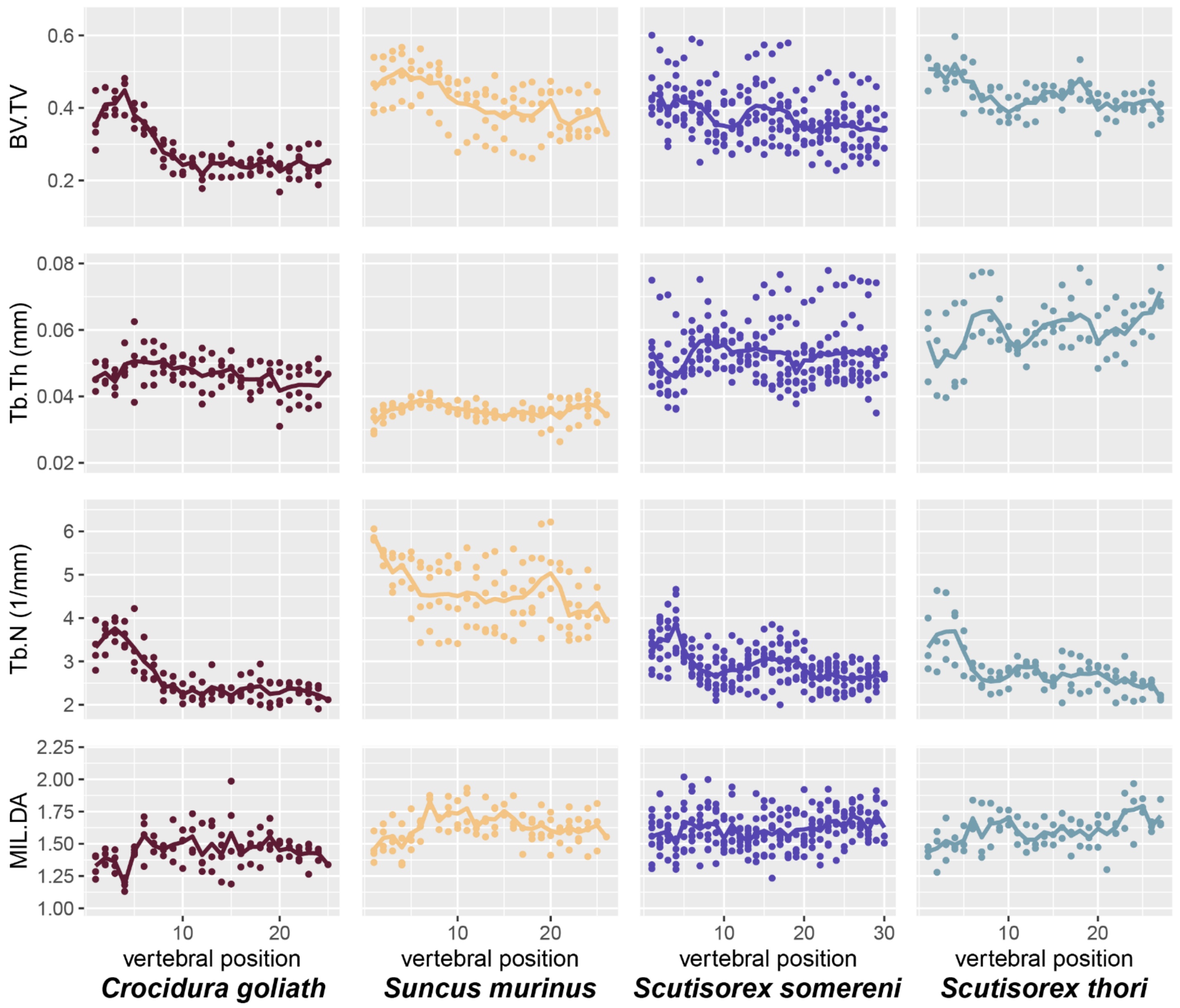

FIGURE S6, Trabecular bone architecture (TBA) metrics for caudal VOIs only. Each point represents the value for the caudal VOI of a single specimen at a given vertebral position.

FIGURE S7, histograms of difference between trabecular bone architecture (TBA) metrics in cranial and caudal vertebral VOIs. X-axes show the magnitude of difference between pairs of VOIs from the same vertebra, with caudal values subtracted from cranial. As a result, negative values show that the caudal VOI has a higher value for the metric, whereas positive values show that the cranial VOI has a higher value. Vertical dashed lines show mean difference between cranial-caudal pairs. Wilcoxon's paired signed rank test comparing cranial and caudal VOIs for all vertebrae yields $p < 2.2e\text{-}16$ for all metrics, indicating a significant difference in TBA in different sections of the bone.

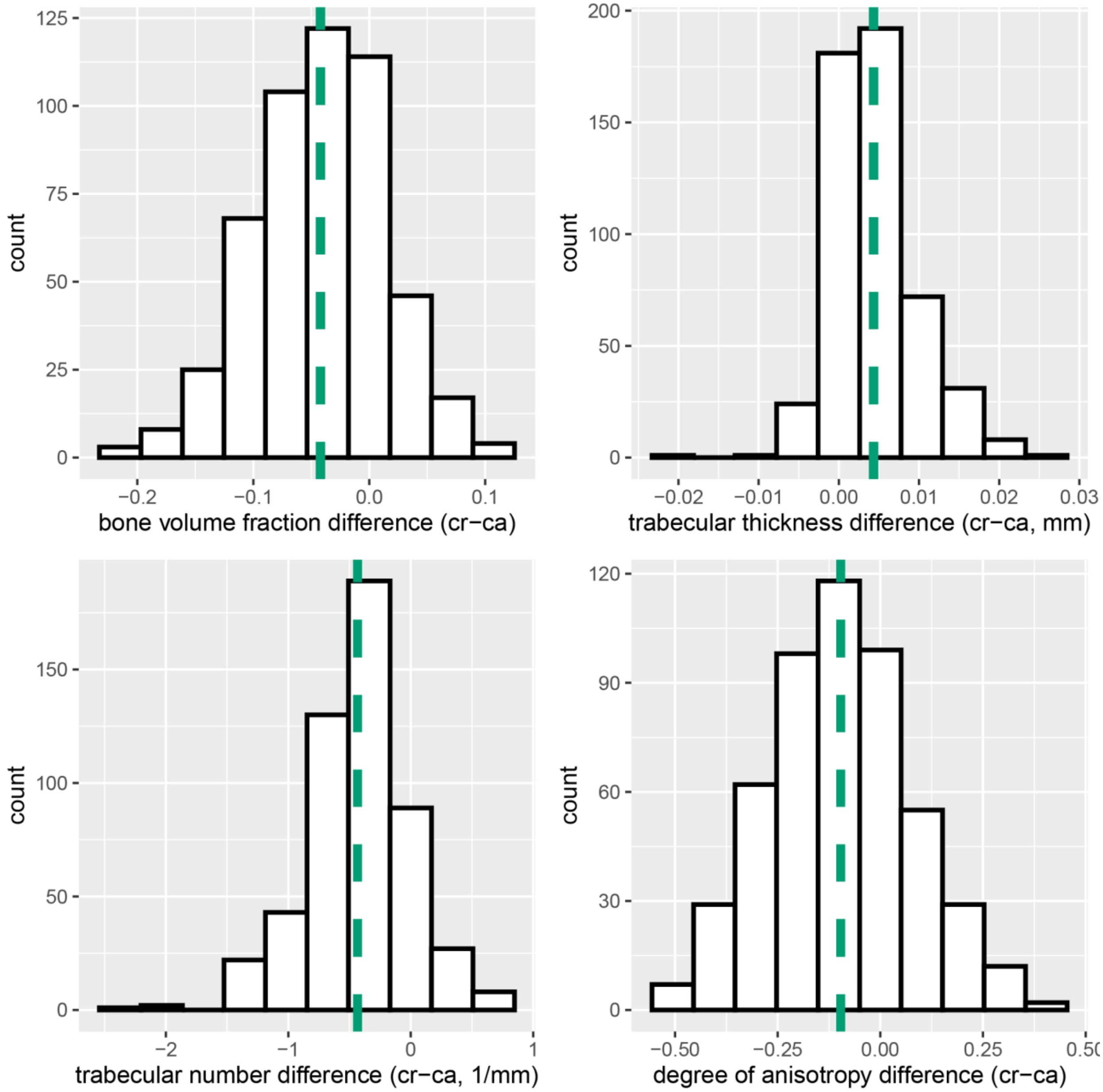

FIGURE S8, region plots for individual specimens of *Crocidura goliath*. Upper plots show regions derived from external morphology, and lower plots show regions derived from trabecular bone architecture (TBA). All region breaks are represented as percentages of the total presacral column length, excluding C01-C02 (axis-atlas complex). Numbers listed are the last position in a given region. See Tables 2-3 for additional details.

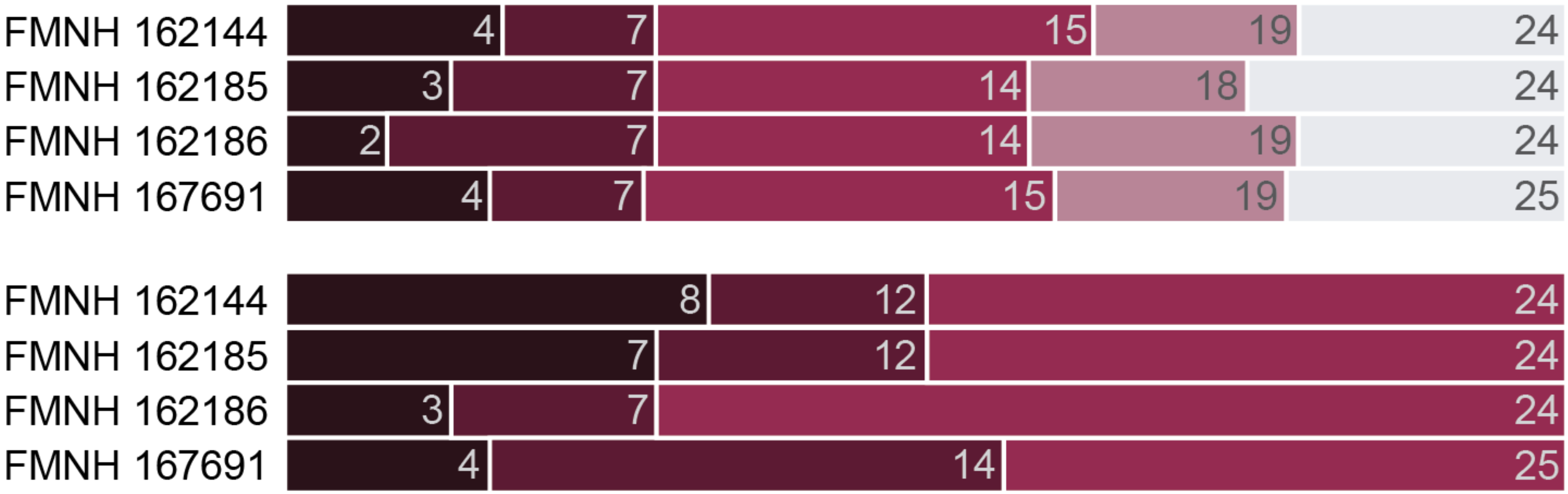

FIGURE S9, region plots for individual specimens of *Suncus murinus*. Upper plots show regions derived from external morphology, and lower plots regions derived from trabecular bone architecture (TBA). All region breaks are represented as percentages of the total presacral column length, excluding C01-C02 (axis-atlas complex). Numbers listed are the last position in a given region. See Tables 2-3 for additional details.

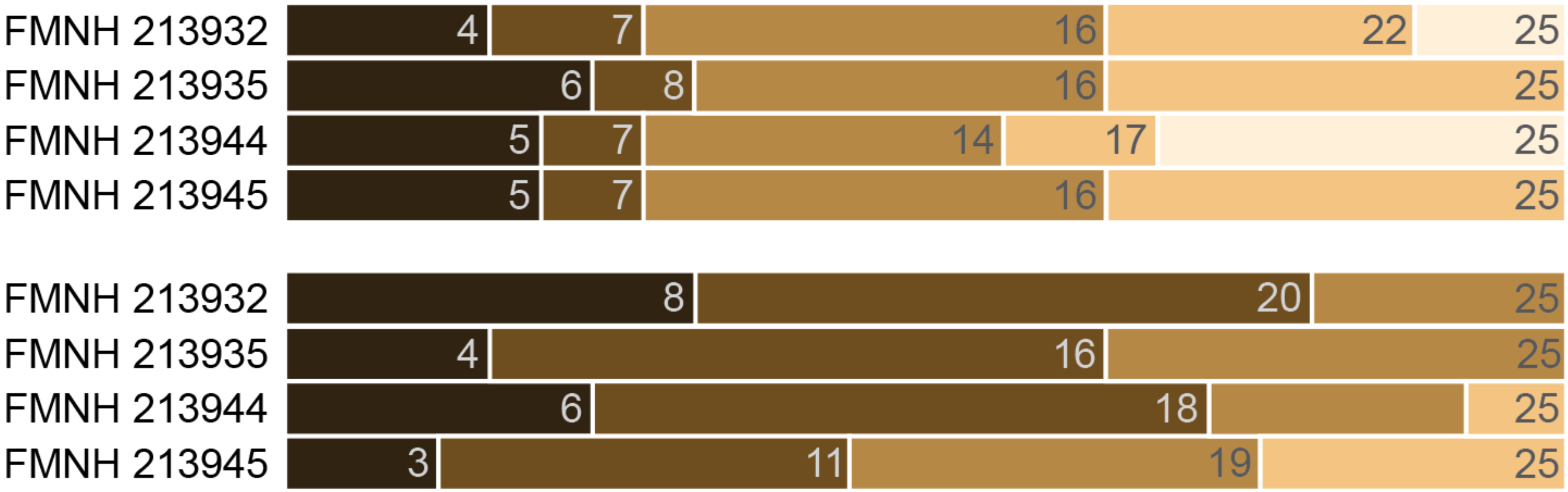

FIGURE S10, region plots for individual specimens of *Scutisorex somereni*. Upper plots show regions derived from external morphology, and lower plots show regions derived from trabecular bone architecture (TBA). All region breaks are represented as percentages of the total presacral column length, excluding C01-C02 (axis-atlas complex). Numbers listed are the last position in a given region. See Tables 2-3 for additional details.

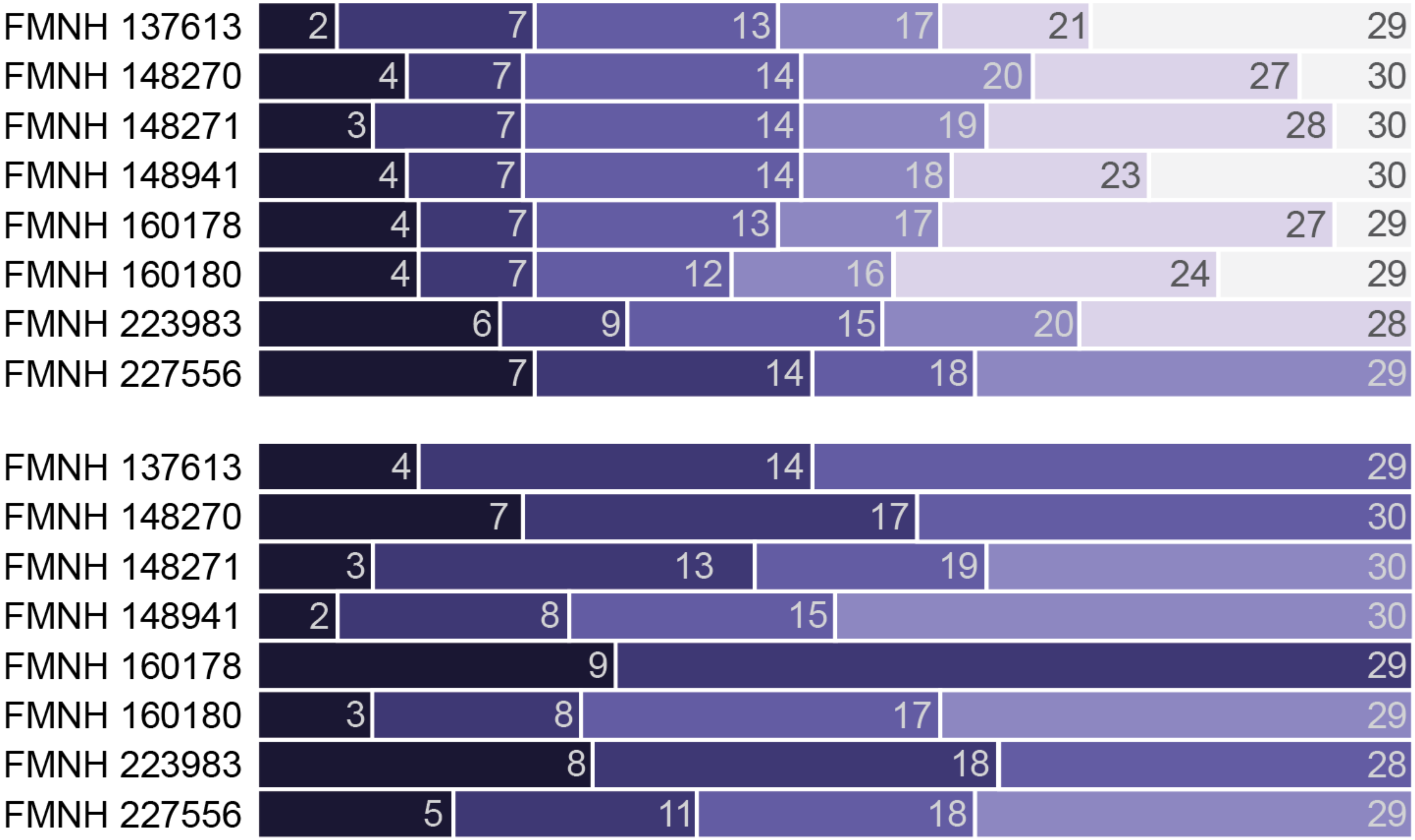

FIGURE S11, region plots for individual specimens of *Scutisorex thori*. Upper plots show regions derived from external morphology, and lower plots show regions derived from trabecular bone architecture (TBA). All region breaks are represented as percentages of the total presacral column length, excluding C01-C02 (axis-atlas complex). Numbers listed are the last position in a given region. See Tables 2-3 for additional details.

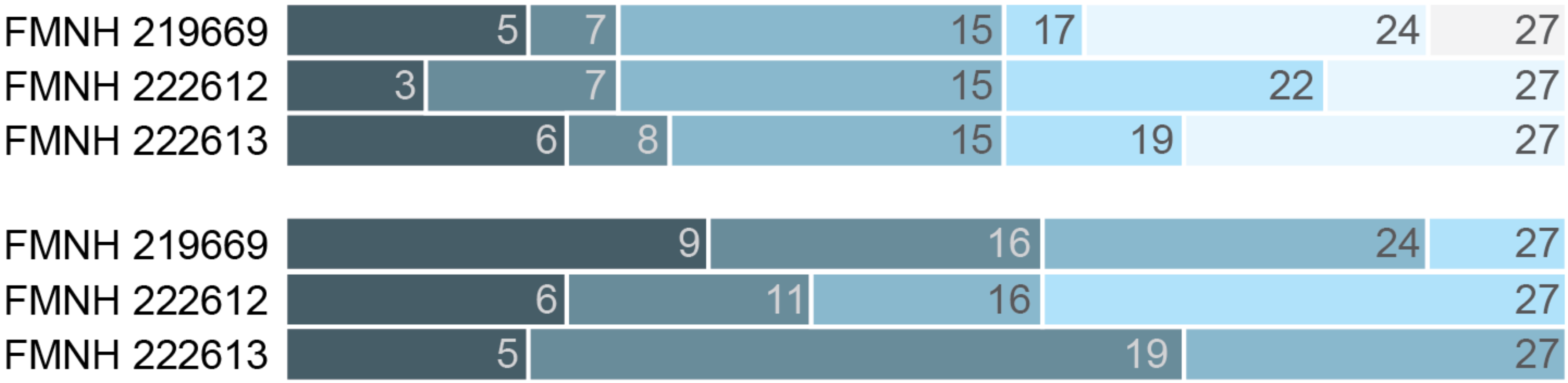

FIGURE S12, boxplots of whole-column heterogeneity for both external morphology and TBA.

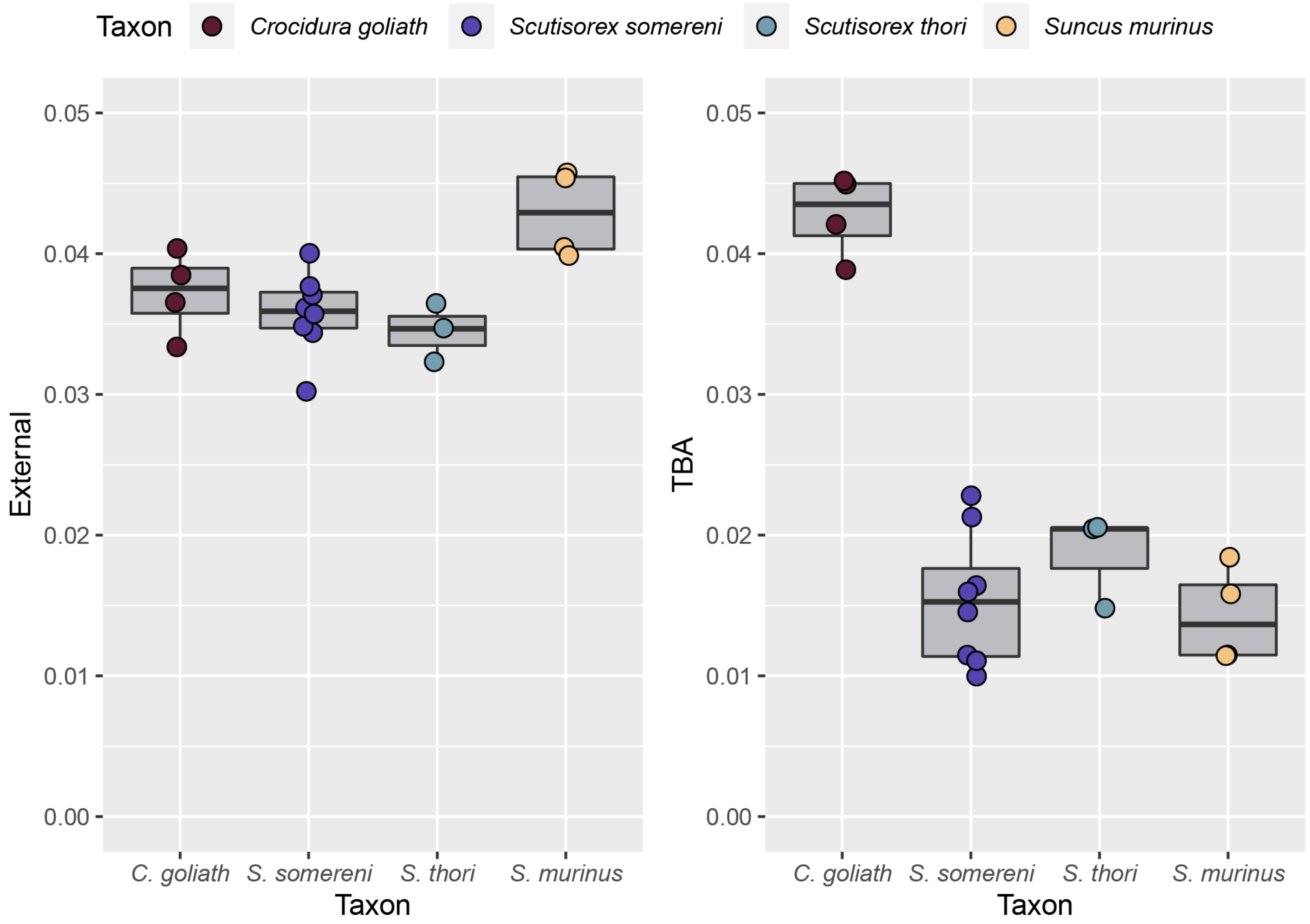

C. ADDITIONAL SUPPLEMENTARY TABLES

TABLE S2, mean relative resolution for all specimens, each taxon, and each specimen. Quant3D relative resolutions and are calculated from Quant3D results of Tb.Th measurements, which are mathematically distinct from those calculated in BoneJ (see Main Text). Values are listed as “mean (range)”. Both cranial and caudal VOIs are included in mean and range values.

Specimen No.	Taxon	Number of VOIs	Tb.Th (mm, Quant3D)	Relative resolution (Quant3D)	Tb.Th (pixels, BoneJ)
**ALL**	**ALL**	1022	0.052 (0.026-0.095)	2.64 (1.65-4.90)	4.38 (2.93-7.37)
**ALL**	*Crocidura goliath*	194	0.048 (0.031-0.063)	2.50 (1.65-3.42)	4.12 (2.93-5.52)
FMNH 162144	*Crocidura goliath*	48	0.048 (0.041-0.056)	2.82 (2.41-3.29)	4.62 (3.99-5.52)
FMNH 162185	*Crocidura goliath*	48	0.047 (0.037-0.063)	2.40 (1.71-3.42)	3.89 (3.04-5.01)
FMNH 162186	*Crocidura goliath*	48	0.048 (0.031-0.063)	2.35 (1.65-3.41)	3.85 (2.93-4.77)
FMNH 167691	*Crocidura goliath*	50	0.048 (0.038-0.062)	2.45 (1.82-3.33)	4.14 (3.17-5.40)
**ALL**	*Suncus murinus*	198	0.037 (0.026-0.046)	2.40 (1.75-3.55)	4.07 (3.05-5.50)
FMNH 213932	*Suncus murinus*	50	0.037 (0.033-0.045)	2.15 (1.91-2.61)	3.74 (3.34-4.38)
FMNH 213935	*Suncus murinus*	48	0.037 (0.030-0.045)	2.24 (1.75-2.92)	3.85 (3.05-4.88)
FMNH 213944	*Suncus murinus*	50	0.038 (0.029-0.046)	2.69 (2.01-3.55)	4.45 (3.53-5.50)
FMNH 213945	*Suncus murinus*	50	0.036 (0.026-0.042)	2.51 (1.91-2.93)	4.21 (3.35-4.74)
**ALL**	*Scutisorex somereni*	468	0.055 (0.035-0.090)	2.59 (1.76-3.90)	4.36 (3.03-6.35)
FMNH 137613	*Scutisorex somereni*	58	0.049 (0.036-0.059)	2.42 (1.78-2.91)	4.09 (3.15-4.79)
FMNH 148270	*Scutisorex somereni*	60	0.053 (0.036-0.067)	2.56 (1.76-3.24)	4.33 (3.03-5.49)
FMNH 148271	*Scutisorex somereni*	60	0.052 (0.041-0.069)	2.50 (1.96-3.33)	4.25 (3.31-5.59)
FMNH 148941	*Scutisorex somereni*	60	0.050 (0.039-0.063)	2.41 (1.91-3.02)	4.15 (3.26-4.49)
FMNH 160178	*Scutisorex somereni*	58	0.052 (0.035-0.062)	2.51 (1.84-3.21)	4.22 (3.40-5.21)
FMNH 160180	*Scutisorex somereni*	58	0.052 (0.038-0.060)	2.71 (2.01-3.17)	4.47 (3.61-5.12)
FMNH 223983	*Scutisorex somereni*	56	0.060 (0.042-0.074)	2.71 (1.83-3.34)	4.50 (3.33-5.48)
FMNH 227556	*Scutisorex somereni*	58	0.073 (0.058-0.090)	2.93 (2.25-3.90)	4.86 (3.86-6.35)
**ALL**	*Scutisorex thori*	162	0.064 (0.040-0.095)	3.22 (1.92-4.90)	5.17 (3.42-7.37)
FMNH 219669	*Scutisorex thori*	54	0.061 (0.040-0.084)	3.12 (2.03-4.31)	5.07 (3.56-6.50)
FMNH 222612	*Scutisorex thori*	54	0.070 (0.056-0.095)	3.52 (2.60-4.90)	5.53 (4.19-7.37)
FMNH 222613	*Scutisorex thori*	54	0.061 (0.043-0.078)	3.02 (1.92-4.69)	4.91 (3.42-7.38)

TABLE S3, mean magnitude difference between TBA metrics in cranial and caudal VOIs. See Figure S7 for distributions.

	BV.TV	Tb.Th (mm)	Tb.N (1/mm)	MIL.DA
C. goliath	0.070	0.0031	0.47	0.15
S. murinus	0.054	0.0027	0.69	0.15
S. somereni	0.039	0.0062	0.39	0.17
S. thori	0.085	0.0078	0.56	0.17
ALL	0.055	0.0052	0.49	0.16
